# Host Transcriptional Response to Influenza and Other Acute Respiratory Viral Infections – A Prospective Cohort Study

**DOI:** 10.1371/journal.ppat.1004869

**Published:** 2015-06-12

**Authors:** Yijie Zhai, Luis M. Franco, Robert L. Atmar, John M. Quarles, Nancy Arden, Kristine L. Bucasas, Janet M. Wells, Diane Niño, Xueqing Wang, Gladys E. Zapata, Chad A. Shaw, John W. Belmont, Robert B. Couch

**Affiliations:** 1 Department of Molecular and Human Genetics, Baylor College of Medicine, Houston, Texas, United States of America; 2 Children’s Nutrition Research Center, Baylor College of Medicine, Houston, Texas, United States of America; 3 Laboratory of Systems Biology, Division of Intramural Research (DIR), National Institute of Allergy and Infectious Diseases (NIAID), National Institutes of Health, Bethesda, Maryland, United States of America; 4 Department of Medicine, Baylor College of Medicine, Houston, Texas, United States of America; 5 Department of Molecular Virology and Microbiology, Baylor College of Medicine, Houston, Texas, United States of America; 6 Department of Microbial and Molecular Pathogenesis, Texas A&M University System Health Science Center, College Station, Texas, United States of America; 7 Department of Pediatrics, Baylor College of Medicine, Houston, Texas, United States of America; Icahn School of Medicine at Mount Sinai, UNITED STATES

## Abstract

To better understand the systemic response to naturally acquired acute respiratory viral infections, we prospectively enrolled 1610 healthy adults in 2009 and 2010. Of these, 142 subjects were followed for detailed evaluation of acute viral respiratory illness. We examined peripheral blood gene expression at 7 timepoints: enrollment, 5 illness visits and the end of each year of the study. 133 completed all study visits and yielded technically adequate peripheral blood microarray gene expression data. Seventy-three (55%) had an influenza virus infection, 64 influenza A and 9 influenza B. The remaining subjects had a rhinovirus infection (N = 32), other viral infections (N = 4), or no viral agent identified (N = 24). The results, which were replicated between two seasons, showed a dramatic upregulation of interferon pathway and innate immunity genes. This persisted for 2-4 days. The data show a recovery phase at days 4 and 6 with differentially expressed transcripts implicated in cell proliferation and repair. By day 21 the gene expression pattern was indistinguishable from baseline (enrollment). Influenza virus infection induced a higher magnitude and longer duration of the shared expression signature of illness compared to the other viral infections. Using lineage and activation state-specific transcripts to produce cell composition scores, patterns of B and T lymphocyte depressions accompanied by a major activation of NK cells were detected in the acute phase of illness. The data also demonstrate multiple dynamic gene modules that are reorganized and strengthened following infection. Finally, we examined pre- and post-infection anti-influenza antibody titers defining novel gene expression correlates.

## Author Summary

Gene expression profiling of human blood cells might uncover the complex dynamics of host response to ARIs such as pandemic H1N1. However, only limited data are available on the system level response to naturally acquired infections. To understand the molecular bases and network orchestration of host responses, we prospectively enrolled 1610 healthy adults in the fall of 2009 and 2010, followed the subjects with influenza-like illness (N = 133) for 3 weeks, and examined changes in their peripheral blood gene expression. We discovered distinct phases of the host response spanning 6 days after infection, and identified genes that differentiate influenza from non-influenza virus infection. We then moved the focus from gene expression patterns to gene co-expression patterns. We detected gene modules that are related to core features of regulatory networks and found a substantial increase in the connectivity of the influenza responsive genes. Finally, we identified a molecular signature that correlated significantly with antibody response to pH1N1 virus. Taken together, our findings offer insights into the molecular mechanisms underlying host response to influenza virus infection, and provide a valuable foundation for investigation of the global coordinated responses to ARIs. Molecular correlates of the immune response suggest targets for intervention and improved vaccines.

## Introduction

Influenza viruses are highly contagious respiratory pathogens that cause about three to five million cases of severe illness, and about 250 000 to 500 000 deaths worldwide each year [1]. In the US, influenza affects an estimated 5% to 20% of the population yearly [2], and is responsible for an average of 3.1 million hospitalized days, and 31.4 million outpatient visits. Direct medical costs are estimated to be at least $10.4 billion annually [3]. A new influenza virus appeared in Mexico and the United States in April 2009 and caused extensive outbreaks of influenza in the population. The virus was promptly identified as a swine-like influenza A (H1N1) virus and shown to be a triple reassortant virus containing genes from swine, human, and avian influenza A viruses [4]. Pandemic swine influenza (pH1N1) peaked in the United States in October 2009, with minimal activity during the subsequent winter period of influenza and reappeared during the winter of 2010–2011. Our recent studies showed that preexisting antibody to the seasonal A/H1N1 virus reduced pH1N1 influenza virus infection and illness in healthy young adults [5, 6].

Complex coordinated responses are triggered in the host following an acute respiratory viral infection. Many aspects of host-pathogen interactions after influenza infection have been studied [7–12]. Blood transcriptome profiling provides a ‘snap shot’ of the systematic host immune networks, as blood circulates throughout the body, carrying naive and educated immune cells, whose transcriptional activity can be influenced by environmental stimuli such as a respiratory virus illness [13]. Transcriptional signatures have been described in the context of ARIs caused by different etiological agents, including influenza, rhinovirus (HRV), and respiratory syncytial virus (RSV), as well as by influenza vaccination [14–23]. These studies have shown that blood gene expression signatures are distinctive for individuals with infection-induced ARI. ARI gene expression signatures show highly significant enrichment for transcripts encoding proteins involved in interferon signaling and pattern recognition induced innate immunity responses [14, 16].

Transcriptome analysis in influenza-infected mouse lungs has revealed distinct phases of the host response extending over at least a two month period after infection [20]. In previously reported studies in humans with ARI, transcriptional profiling was only performed on RNA samples collected either at a single timepoint of peak symptoms, or within the initial 2 to 3 days of hospitalization. The dynamic changes over the entire time course of naturally acquired infection and illness in humans are less clear. Experimentally induced influenza infection has been used to obtain information about changes in temporal gene expression [14, 21]. Huang et al made important observations about the differences in response between asymptomatic and symptomatic individuals. These studies, however, were limited by sample size and could not contrast other common respiratory virus agents with influenza. Menachery et al reported a contrasting gene signature between pH1N1 and coronavirus infected airway epithelial cells [22]. The genes they investigated were limited to interferon-stimulated genes. Studies to characterize the temporal dynamics of the systemic transcriptional response to ARI in humans are necessary to better understand the biology of infection, the host response and occurrence of disease. Furthermore, serum antibody responses to influenza virus infection have large inter-individual variation [5, 6]. Several influenza vaccine studies showed genes that play a role in antigen presentation and T cell recognition are associated with influenza vaccine-induced antibodies [19, 23–25]. Whether the same sets of genes contribute to the variation in antibody response to naturally acquired influenza infection is not known.

Approaches to uncover the modular organization and function of transcriptional systems have shown promise in facilitating functional interpretation and discovering biological networks. These models have been successfully applied in several biological contexts [26–28]. Weighted Gene Co-expression Network Analysis (WGCNA) group sets of genes with similar transcriptional patterns together to form a transcriptional module. Since the probability for multiple transcripts to follow a complex pattern of expression across all the samples by chance is low, such sets of genes should constitute coherent and biologically meaningful transcriptional units [29, 30]. Recently developed differential co-expression analysis goes beyond identification of differentially expressed genes (DEGs) or pathways to identify differential co-expression pattern [31–33]. Under the premise that pairwise correlations between gene expression levels result from regulatory relationships among the genes, major changes in co-expression patterns between two conditions may indicate dysfunctional regulatory systems in disease.

The clinical, virological and immunological results of our prospective study of ARI in a young adult population that included influenza and other known pathogenic viruses have been reported [5, 6]. Using genome wide transcript profiling we provide evidence in this report for three distinct phases of response among those persons with ARI: a) acute systemic activation of the innate response; b) recovery with extensive cell repair and proliferation; and 3) restoration of baseline gene expression patterns. These results provide new transcriptional correlates for the evolution of ARI. The results indicate a central role for interferon and innate immunity in the acute phase of the illness. The recovery phase has not been well characterized previously and suggests new avenues for understanding the restoration of biological system equilibrium after infection and illness.

## Results

### Etiology and demographics of the subjects with ARIs

1610 healthy adults were prospectively enrolled before the influenza seasons of 2009–10 and 2010–11. Of these, 142 (8.8%) who subsequently developed a moderate influenza-like illness were enrolled for follow up; none met the criteria for severe respiratory disease. Of the 142 enrolled ill subjects, 133 reported for all scheduled study visits and had technically adequate gene expression data (vide infra). Table 1 summarizes the infection and demographic data for these 133 subjects. Viral culture and RT-PCR for respiratory viruses indicated that 64 were infected with influenza A virus, and 9 were infected with influenza B virus. Infection with a rhinovirus, respiratory syncytial virus (RSVA/RSVB), coronavirus (OC43, 229E, NL63, HKU1), or enterovirus (Entero) was also detected in a number of the subjects with influenza-like symptoms. There were 24 individuals with an influenza-like illness for whom no virus was identified. The subjects were predominantly European-Americans (80.5%), consistent with the study area population.

**Table 1:** Viral infections and demographic information of the 133 subjects with influenza-like illness enrolled at Texas A&M University in Fall 2009 and 2010 from whom microarray expression data were available.

	2009 Cohort	2010 Cohort	Total	% of Subjects with ARI
Infections				
Influenza A	**24**	**21**	**45**	**33.8**
Influenza A + HRV	**6**	**10**	**16**	**12.0**
Influenza A + RSVB	**0**	**1**	**1**	**<1**
Influenza A + OC43	**0**	**1**	**1**	**<1**
Influenza A + 229E	**1**	**0**	**1**	**<1**
Influenza B	**0**	**4**	**4**	**3.0**
Influenza B + HRV	**0**	**5**	**5**	**3.8**
HRV	**19**	**6**	**25**	**18.8**
HRV + RSVA	**0**	**1**	**1**	**<1**
HRV + RSVB	**0**	**1**	**1**	**<1**
HRV + NL63	**0**	**1**	**1**	**<1**
HRV + HKU1	**2**	**1**	**3**	**2.3**
HRV + Entero	**1**	**0**	**1**	**<1**
RSVA	**1**	**0**	**1**	**<1**
NL63	**0**	**1**	**1**	**<1**
HKU1	**1**	**0**	**1**	**<1**
Entero	**1**	**0**	**1**	**<1**
Unknown	**17**	**7**	**24**	**18.0**
Ethnicity/Race				
White	**58**	**49**	**107**	**80.5**
Indian-American	**10**	**6**	**16**	**12.0**
African-American	**5**	**3**	**8**	**6.0**
Asian	**0**	**2**	**2**	**1.5**

*Influenza A = Influenza Virus type A; Influenza B = Influenza Virus type B; HRV = Human rhinovirus; RSVA = Respiratory Syncytial Virus type A; RSVB = Respiratory Syncytial Virus type B; OC43 = Human coronavirus OC43; 229E = Human coronavirus 229E; NL63 = Human coronavirus NL63; HKU1 = Human coronavirus HKU1; Entero = Enterovirus; Unknown: Our tests did not detect one of the viruses sought.

### Global gene expression profile for influenza infection in adults

**Fig 1:**
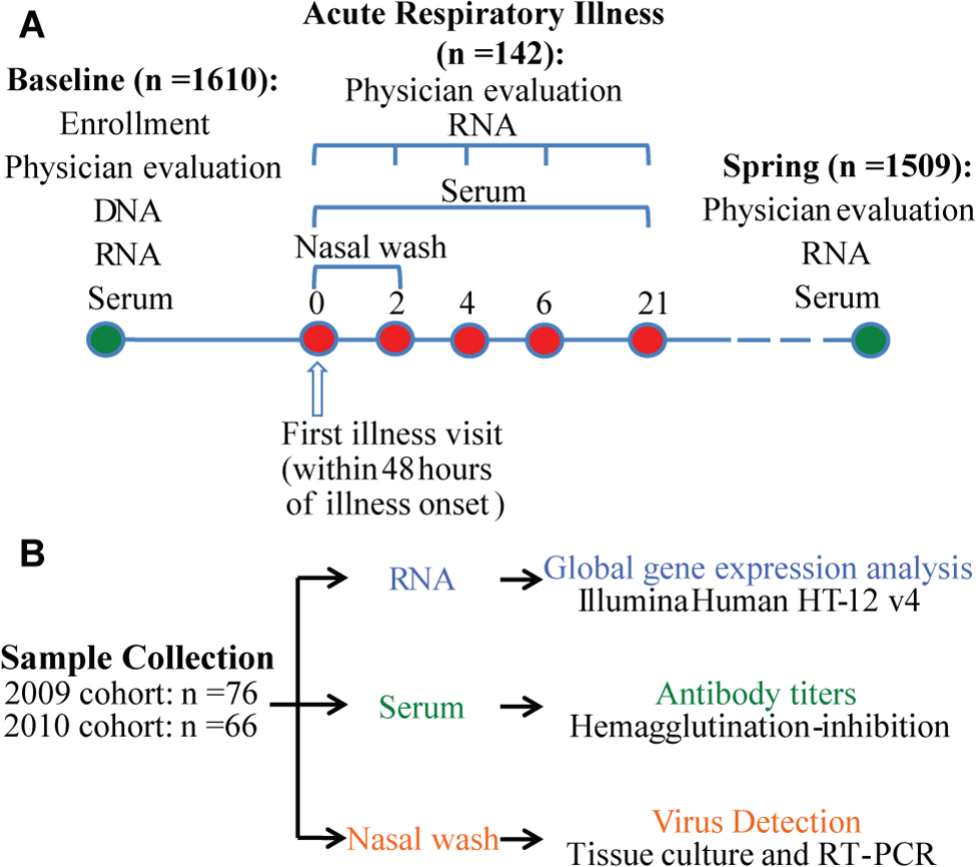
Study design and analysis scheme. **(A)** 1610 individuals were enrolled before the influenza season in 2009 and 2010. Peripheral blood samples and nasal secretion samples were collected from each subject at the beginning of enrollment for influenza antibody tests. Genomic DNA and whole blood RNA were obtained from blood samples. Those subjects who became ill with influenza-like symptoms (N = 142) were seen within 48 hours of onset and 2, 4, and 6 days later for repeat evaluation, specimen collections, and medical care and 21 days later for collection of convalescent specimens. Nasal wash samples were collected for virus detection on day 0 and day 2. 1509 of the enrolled subjects completed the study and were called back in the spring of the next year for collecting whole blood RNA, serum and nasal wash samples. **(B)** Sample size and data generation.

We analyzed the global gene expression profiles of peripheral whole blood in the 133 adults with an ARI at up to seven time points before, during, and after the occurrence of illness (Fig 1A and 1B). Because the subjects were enrolled prospectively we had control samples taken from the same subject before occurrence of illness (baseline samples). A total of 890 microarray analyses were completed. Samples which failed QC were excluded from the analyses (N = 10), leaving 880 high quality arrays from which the subsequent analysis was conducted. Differential expression analyses for each day, compared to the baseline were then stratified by viral agent. We first analyzed the gene expression profiles in 49 subjects from whom an influenza virus infection was identified. The 24 subjects with influenza A virus infection in the 2009 cohort were used as a discovery group, and the consistency of differential expressed genes was assessed in another 21 influenza A virus infected subjects and 4 influenza B virus infected subjects in the 2010 cohort as a validation group. After performing significance testing with corrections for multiple tests, we detected highly significant expression differences in thousands of transcripts during the period of influenza illness (days 0, 2, 4, 6) in both discovery and validation groups. In contrast, once the subject had clinically recovered there were no significant expression differences detected (day 21, and spring samples).

### A robust and dynamic host transcriptional response to influenza virus infection still present after cell composition changes were taken into account

**Fig 2:**
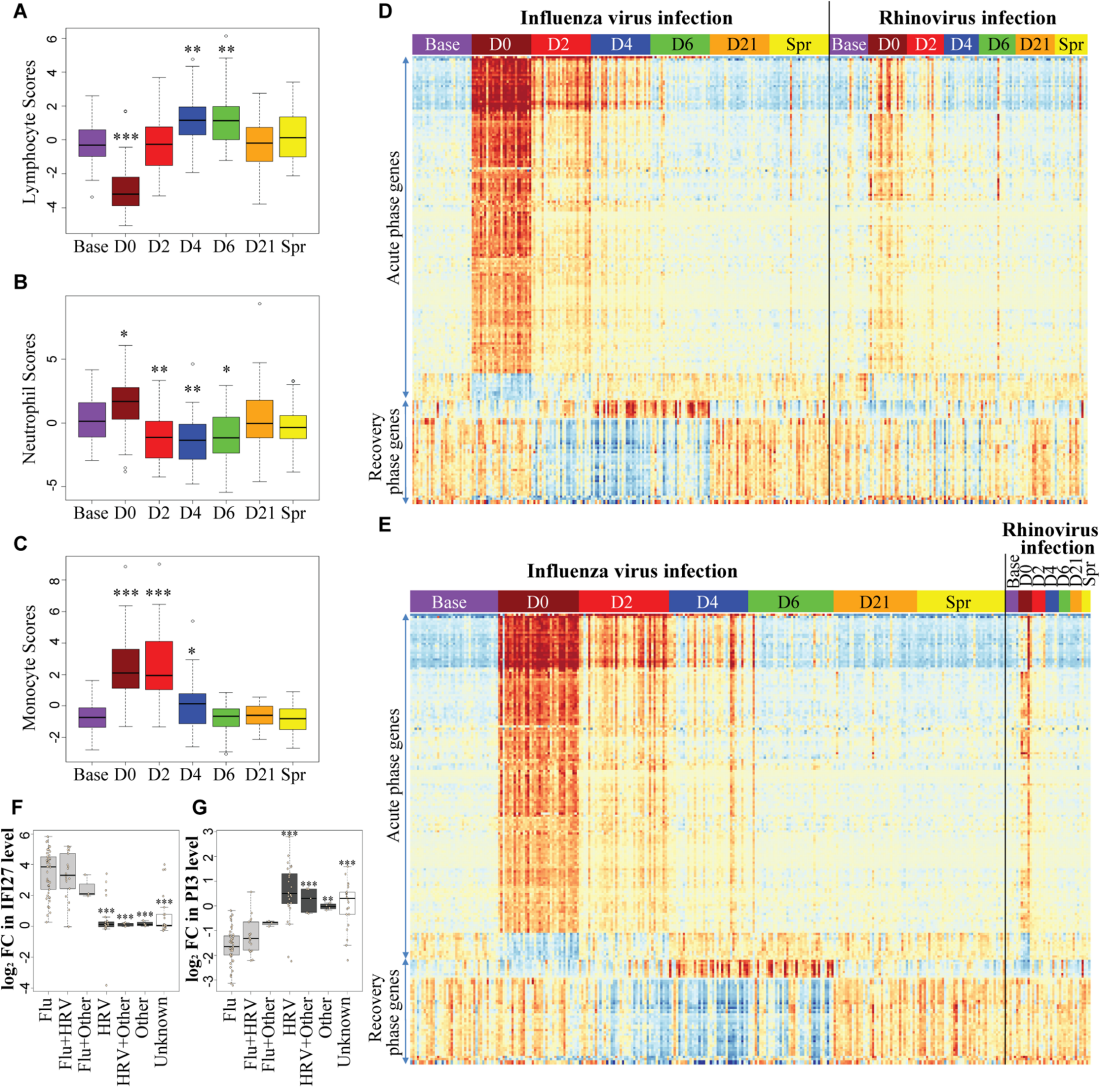
A robust and dynamic host transcriptional response to influenza virus infection. **(A-C)** Peripheral blood cell composition was altered by influenza virus infection. Cell scores for **(A)** lymphocyte, **(B)** neutrophil and **(C)** monocyte were computed for each sample from influenza-infected individuals, by taking the PC1 of normalized expression levels of the lineage-specific gene sets (See S3 Table for the list of lineage specific genes). One-way analysis of variance (ANOVA) was used to determine whether there are significant differences between each illness day and baseline. **(D-E)** Heatmaps demonstrating the time course of the genes showing the most significant pattern of differential expression compared to baseline in patients with influenza virus and/or rhinovirus infection. **(D)** 2009 Cohort, **(E)** 2010 Cohort. Each column corresponds to an individual RNA sample and each row represents the mean-centered, normalized expression values for each of the differentially expressed genes (BH-corrected *P* values <0.05, |log_2_ FC| >1 in both 2009 and 2010 cohorts). Samples were grouped by day and subjects were grouped by infections status (influenza virus infection group includes influenza A, influenza B, influenza A +rhinovirus and influenza B +rhinovirus infections). The transcript order was determined by hierarchical clustering and the order was the same in the two heatmaps. There are three clear phases of transcriptional regulation in response to infection– 1) an acute phase seen on the first day of illness that persisted for 2–4 days; 2) a recovery phase that peaked on day 4 and day 6 after virus infection; 3) restoration of baseline gene expression patterns by day 21. A full list of the 202 transcript probes in the heatmaps and their corresponding genes is provided in S1 Table. **(F-G)** Expression changes of **(F)** *IFI27* and **(G)** *PI3* discriminate between infections with influenza virus and rhinovirus. Fold Changes of *IFI27* and *PI3* were measured in paired day 0 –baseline samples from patients with ARI. Subjects were grouped by infections status. Enterovirus, HKU1, NL63, and RSV infections were grouped together as “Other” virus. One-way ANOVA was used to determine whether there are any significant differences between influenza virus infection groups (Grey) and each non-influenza virus group (Black). ***, P <0.001; **, P <0.005; *, P <0.01.

Since blood is a complex tissue, changes in transcript abundance can be attributed to either transcriptional regulation or changes in the composition of leukocyte populations. To “deconvolute” these two phenomena, we computed a cell score derived from the expression profile of each sample using a composite of lymphocyte, neutrophil or monocyte specific transcripts. We found that lymphocyte lineagespecific transcripts were depressed in the acute phase of influenza virus infection, increased above baseline in the recovery phase, and then returned to baseline on day 21 (Fig 2A). An opposite change in neutrophil score was observed (Fig 2B). Expression levels of monocyte markers were increased in the acute phase and returned to baseline on day 6 (Fig 2C). These changes in established lineage markers of the broad cell populations probably track the changes in cell composition in the peripheral blood. The changes in lymphocyte and neutrophil proportions we predicted “*in silico*” are consistent with the changes described in experimental human challenges with influenza virus [34, 35]. Changes in lineage composition could explain part of the differential gene expression observed during the infection. We therefore recomputed the differential expression analysis using the lymphocyte and neutrophil scores as covariates in a series of contrasts focusing on days 0 to day 6 compared to baseline. Although the *p-values* were slightly increased, the rank ordering of genes showing highly specific differential expression was nearly identical (Tables 2–5). This indicates that while cell composition does affect estimates of total transcript abundance, the most important component of the differential expression arises from changes in transcript abundance within those populations. On a global scale, changes in the host transcriptomes were observed from the first day of illness through day 6 evaluations. A total of 4,706 differentially expressed genes (DEGs) (BH-corrected *P* values <0.05 in both cohorts) were identified over the course of 6 days of influenza virus illness (S1A Fig). 2119 transcripts, corresponding to 1421 genes, were responsive to the infectious stimulus on day 0 (day 1 or 2 of illness). The number of DEGs peaked at day 4. On day 6, only a small number (N = 46) of DEGs were newly detected (i.e. DEGs that first appeared on day 6 and were not detected at any time before). 738 out of the 1140 DEGs with |log2 Fold-Change| > 0.3 were first detected on day 0 (S1B Fig).

**Table 2:** Top upregulated genes in the acute phase of influenza virus infection.

Gene Symbol	Gene Name	logFC	adj.*P* Value	After deconvolution	
				logFC	adj.*P* Value
**IFI44L**	interferon-induced protein 44-like	4.21	1.80E-35	4.18	6.29E-31
**ISG15**	ISG15 ubiquitin-like modifier	4.47	6.51E-26	4.14	9.86E-21
**IFIT1**	interferon-induced protein with tetratricopeptide repeats 1	4.13	5.54E-23	4.13	8.72E-22
**IFI27**	interferon, alpha-inducible protein 27	3.08	5.09E-12	4.09	2.27E-10
**IFITM3**	interferon induced transmembrane protein 3	3.70	1.93E-22	3.99	2.21E-15
**RSAD2**	radical S-adenosyl methionine domain containing 2	4.12	1.14E-25	3.80	2.81E-23
**LY6E**	lymphocyte antigen 6 complex, locus E	3.42	2.44E-29	3.73	2.47E-26
**MX1**	myxovirus (influenza virus) resistance 1, interferon-inducible protein p78 (mouse)	3.65	2.10E-24	3.63	3.97E-22
**IFIT3**	interferon-induced protein with tetratricopeptide repeats 3	3.73	3.18E-25	3.62	8.81E-22
**IFI6**	interferon, alpha-inducible protein 6	3.48	8.64E-24	3.58	1.54E-23
**HERC5**	hect domain and RLD 5	3.86	1.48E-22	3.42	5.50E-19
**EPSTI1**	epithelial stromal interaction 1 (breast)	3.47	1.96E-26	3.30	2.71E-24
**IFIT2**	interferon-induced protein with tetratricopeptide repeats 2	3.51	4.98E-21	3.19	1.11E-18
**IFI44**	interferon-induced protein 44	3.29	2.44E-29	3.19	2.09E-24
**OAS2**	2'-5'-oligoadenylate synthetase 2, 69/71kDa	3.37	1.25E-27	3.07	2.81E-23
**OAS1**	2'-5'-oligoadenylate synthetase 1, 40/46kDa	3.16	1.14E-25	2.88	2.85E-20
**IRF7**	interferon regulatory factor 7	3.20	6.51E-26	2.86	4.14E-19
**OAS3**	2'-5'-oligoadenylate synthetase 3, 100kDa	3.24	5.86E-25	2.80	9.28E-21
**OASL**	2'-5'-oligoadenylate synthetase-like	3.16	5.41E-20	2.72	5.75E-16
**MT1A**	metallothionein 1A	3.24	1.21E-19	2.71	7.04E-13
**XAF1**	XIAP associated factor 1	2.80	1.78E-31	2.68	1.10E-24
**GBP1**	guanylate binding protein 1, interferon-inducible	2.95	6.14E-22	2.64	2.15E-14
**STAT2**	signal transducer and activator of transcription 2, 113kDa	2.49	1.39E-22	2.36	2.99E-15
**LAP3**	leucine aminopeptidase 3	2.44	3.73E-21	2.28	5.16E-14
**GBP5**	guanylate binding protein 5	2.48	5.77E-20	2.11	4.16E-12

**Table 3:** Top downregulated genes in the acute phase of influenza virus infection.

Gene Symbol	Gene Name	logFC	adj.*P* Value	After deconvolution	
				logFC	adj.*P* Value
**PI3**	peptidase inhibitor 3, skin-derived	-1.51	6.40E-10	-2.11	4.09E-09
**ALPL**	alkaline phosphatase, liver/bone/kidney	-0.87	1.41E-03	-1.18	4.73E-04
**RPL3**	ribosomal protein L3	-1.30	9.50E-14	-1.05	4.73E-09
**EIF3L**	eukaryotic translation initiation factor 3, subunit L	-1.12	3.25E-15	-1.02	5.08E-13
**RPS4X**	ribosomal protein S4, X-linked	-1.21	5.55E-13	-1.01	1.18E-06
**RPL23AP64**	ribosomal protein L23a pseudogene 64	-0.85	1.39E-04	-1.00	6.99E-04
**RPS8**	ribosomal protein S8	-1.16	2.13E-13	-0.99	1.80E-09
**MME**	membrane metallo-endopeptidase	-0.89	6.06E-07	-0.99	1.07E-05
**RPL5**	ribosomal protein L5	-1.07	6.26E-13	-0.98	1.54E-08
**TXNDC12**	thioredoxin domain containing 12 (endoplasmic reticulum)	-1.03	1.68E-20	-0.98	1.45E-16
**RPS3**	ribosomal protein S3	-1.20	3.87E-15	-0.96	3.11E-08
**RPS27A**	ribosomal protein S27a	-0.95	4.55E-10	-0.96	1.93E-07
**RPS5**	ribosomal protein S5	-1.24	8.98E-14	-0.93	1.80E-09
**EMR3**	egf-like module containing, mucin-like, hormone receptor-like 3	-0.87	1.95E-12	-0.93	7.91E-09
**LOC729021**	hypothetical protein LOC729021	-0.51	3.25E-04	-0.93	1.56E-06
**RPL7A**	ribosomal protein L7a	-0.94	5.26E-11	-0.90	1.68E-10
**SGK1**	serum/glucocorticoid regulated kinase 1	-1.11	1.17E-15	-0.88	9.67E-08
**EIF4B**	eukaryotic translation initiation factor 4B	-1.07	8.65E-15	-0.87	4.71E-14
**CMTM2**	CKLF-like MARVEL transmembrane domain containing 2	-0.52	8.21E-05	-0.87	1.73E-06
**EEF1G**	eukaryotic translation elongation factor 1 gamma	-1.10	5.64E-16	-0.86	4.25E-08
**RPL4**	ribosomal protein L4	-1.09	1.09E-14	-0.86	1.68E-07
**RPS6**	ribosomal protein S6	-1.09	4.48E-12	-0.86	6.21E-07
**RPS28**	ribosomal protein S28	-0.80	1.42E-03	-0.86	1.39E-03
**KLRB1**	killer cell lectin-like receptor subfamily B, member 1	-0.71	6.90E-09	-0.85	1.84E-06
**RPL14**	ribosomal protein L14	-0.47	5.71E-04	-0.85	8.26E-07

**Table 4:** Top upregulated genes in the recovery phase of influenza virus infection.

Gene Symbol	Gene Name	logFC	adj.*P* Value	After deconvolution	
				logFC	adj.*P* Value
**IFI27**	interferon, alpha-inducible protein 27	2.80	1.74E-14	2.84	6.01E-12
**IGJ**	immunoglobulin J polypeptide, linker protein for immunoglobulin alpha and mu polypeptides	1.35	3.36E-05	1.51	7.66E-05
**IGLL1**	immunoglobulin lambda-like polypeptide 1	1.16	5.71E-05	1.45	3.72E-05
**LOC652694**	similar to Ig kappa chain V-I region HK102 precursor	1.40	4.45E-05	1.42	5.30E-04
**TXNDC5**	thioredoxin domain containing 5 (endoplasmic reticulum)	0.98	2.62E-04	1.06	1.55E-04
**LY6E**	lymphocyte antigen 6 complex, locus E	0.95	1.02E-07	0.98	8.20E-06
**EPSTI1**	epithelial stromal interaction 1 (breast)	0.87	2.78E-06	0.87	1.55E-04
**HSP90B1**	heat shock protein 90kDa beta (Grp94), member 1	0.82	8.25E-06	0.73	6.36E-04
**MZB1**	marginal zone B and B1 cell-specific protein	0.56	3.24E-03	0.72	3.28E-04
**RGS18**	regulator of G-protein signaling 18	0.47	1.14E-03	0.62	4.03E-04
**XBP1**	X-box binding protein 1	0.68	1.45E-05	0.59	3.83E-04
**SEC11C**	SEC11 homolog C (S. cerevisiae)	0.47	7.85E-05	0.56	5.05E-05
**ITGB1**	integrin beta1(fibronectin receptor)	0.62	2.97E-06	0.54	3.23E-04
**IFIT1**	interferon-induced protein with tetratricopeptide repeats 1	0.35	2.44E-02	0.53	1.53E-02
**IFI44L**	interferon-induced protein 44-like	0.54	1.01E-03	0.52	2.96E-03
**IFI44**	interferon-induced protein 44	0.48	5.05E-04	0.49	7.68E-04
**IL8**	interleukin 8	0.42	1.91E-04	0.49	4.94E-04
**GZMA**	granzyme A (granzyme 1, cytotoxic T-lymphocyte-associated serine esterase 3)	0.60	1.23E-05	0.48	1.31E-03
**ARGLU1**	arginine and glutamate rich 1	0.50	1.12E-05	0.48	3.57E-04
**ADD3**	adducin 3 (gamma)	0.49	3.75E-05	0.48	9.41E-04
**PSMA6**	proteasome (prosome, macropain) subunit, alpha type, 6	0.32	9.41E-04	0.47	5.89E-04
**TMEM123**	transmembrane protein 123	0.50	1.70E-05	0.46	6.62E-04
**EVI2A**	ecotropic viral integration site 2A	0.39	6.90E-04	0.45	1.50E-03
**ZRANB2**	zinc finger, RAN-binding domain containing 2	0.45	2.36E-05	0.43	8.58E-04
**ITM2C**	integral membrane protein 2C	0.41	2.09E-03	0.43	2.50E-03

**Table 5:** Top downregulated genes in the recovery phase of influenza virus infection.

Gene Symbol	Gene Name	logFC	adj.*P* Value	After deconvolution	
				logFC	adj.*P* Value
**LOC100131726**	HCC-related HCC-C11_v3	-1.49	1.04E-06	-1.33	1.41E-04
**RNF213**	ring finger protein 213	-1.41	6.96E-06	-1.24	5.44E-04
**RN28S1**	RNA, 28S ribosomal 1	-1.14	1.03E-03	-1.07	6.48E-03
**STRADB**	STE20-related kinase adaptor beta	-1.12	3.81E-05	-0.92	3.03E-03
**EPB42**	erythrocyte membrane protein band 4.2	-1.02	1.49E-06	-0.89	2.37E-04
**TPRA1**	transmembrane protein, adipocyte associated 1	-0.98	1.47E-05	-0.84	7.78E-04
**DPYSL5**	dihydropyrimidinase-like 5	-0.92	2.02E-05	-0.80	1.02E-03
**TESC**	tescalcin	-0.89	8.54E-06	-0.80	5.61E-04
**ADIPOR1**	adiponectin receptor 1	-1.01	5.87E-06	-0.77	1.03E-03
**SPRYD3**	SPRY domain containing 3	-0.90	8.48E-05	-0.77	2.19E-03
**GYPC**	glycophorin C (Gerbich blood group)	-0.81	4.39E-05	-0.73	2.03E-03
**CSDA**	cold shock domain protein A	-0.85	7.02E-05	-0.71	3.59E-03
**ASCC2**	activating signal cointegrator 1 complex subunit 2	-0.83	6.55E-05	-0.71	3.52E-03
**UBXN6**	UBX domain protein 6	-0.82	1.18E-05	-0.71	9.75E-04
**MAP1S**	microtubule-associated protein 1S	-0.81	1.10E-06	-0.71	1.51E-04
**TSPAN5**	tetraspanin 5	-0.79	7.95E-07	-0.70	1.33E-04
**NECAP2**	NECAP endocytosis associated 2	-0.74	2.70E-06	-0.69	1.67E-04
**SLC25A37**	solute carrier family 25, member 37	-1.00	4.95E-06	-0.68	1.14E-03
**LOC729021**	hypothetical protein LOC729021	-0.95	2.62E-07	-0.67	6.45E-05
**MUC6**	mucin 6, oligomeric mucus/gel-forming	-0.76	2.09E-05	-0.66	9.70E-04
**HAGH**	hydroxyacylglutathione hydrolase	-0.73	1.01E-04	-0.65	3.34E-03
**TAGLN2**	transgelin 2	-0.79	7.76E-07	-0.64	1.51E-04
**SLC4A1**	solute carrier family 4, anion exchanger, member 1 (erythrocyte membrane protein band 3, Diego blood group)	-0.76	2.41E-04	-0.62	9.94E-03
**LST1**	leukocyte specific transcript 1	-0.82	4.22E-06	-0.60	7.71E-04
**SORL1**	sortilin-related receptor, L(DLR class) A repeats containing	-0.75	7.97E-07	-0.60	4.85E-05

### Subjects with influenza virus infection showed a characteristic three-phase response at the level of the transcript profile

We plotted a heatmap of the 202 transcripts (S1 Table) showing the most significant pattern of differential expression compared to baseline (Fig 2D and 2E), and determined the transcript order by hierarchical clustering. These genes fall into two clusters: 1) genes that were regulated in the acute phase of influenza virus infection, and 2) genes that became differentially expressed at a later time-point (recovery phase). All individuals showed complete recovery to the baseline transcript profile by day 21 after onset of illness. In the acute phase, there was a very large increase in components of the interferon pathway and innate immunity (e.g. *IFI44L*, *IFIT1*, *MX1*, *IFITM3*, *OAS2*, *IFI27* and *IFIT3*, see Table 2), as well as decreased expression of genes involved in translational elongation and protein biosynthesis (e.g. *RPS4X*, *RPS18*, *RPS6*, *RPS8* and *RPL5*, see Table 3). This was most intense on the first day of illness and continued for 2–4 days. This phase was followed by a characteristic recovery phase in which there was a transition to genes involved in antigen binding and antibody secretion (*IGJ*, *LOC652694*, *IGLL1* and *MZB1*, see Table 4) and genes regulating cell morphogenesis (*STRADB*, *DPYSL5*, *EPB42*, *LST1* and *MAP1S*, see Table 5). Inter-individual variations in the magnitude of transcriptional response at each phase were observed (S2 Fig), and greater variations were seen at the times when the transcriptional responses were strong. The expression profiles for individuals infected with Influenza A and B virus were indistinguishable. Likewise individuals infected with both influenza and rhinovirus were not different from those infected with influenza virus alone. No statistically significant differences in expression of any transcripts were identified that marked the mixed infection group.

### A shared host transcriptional response to acute respiratory viral infections

Although there were significant differences in gene expression between the non-influenza virus infection group (e.g. HRV, RSV, coronavirus and enterovirus) and the influenza group (Fig 2D and 2E, S3 Fig), the patterns of the three-phase transcriptional responses were nearly identical, and the differential expression was largely explained by differences in the magnitude of effect. This indicates that the host response to acute respiratory viral infection, despite the distinctive biology of these diverse viruses, is largely conserved. By performing differential expression analysis comparing influenza virus and rhinovirus infection group (Criteria for DEGs were BH-corrected *P* values < 0.0001), we found that comparing to rhinovirus, influenza virus infection and illnesses induced a larger magnitude and longer duration of activation of interferon signaling pathway, and a greater depression in translation and protein biosynthesis (S4A and S4B Fig, S2 Table). Some of the DEGs encode kinase or kinase inhibitor (e.g. *MAPK1*, *PAK2*, *CDKN1A* and *CDKN1B*, S4C Fig). Several protein phosphatase encoding genes were also differentially expressed, such as *PPM1M*, *PPP2R4*, *PPP3CA*, *etc*. However, the magnitudes of differential expression in these genes were small and there were only two transcripts showed |log_2_ fold-change| >1.5: *IFI27* was consistently upregulated in the influenza virus group on days 0–6 but not upregulated in the rhinovirus or other infection groups (Fig 2F); in addition, *PI3* was consistently downregulated in the influenza-infected individuals but not in the other groups (Fig 2G). The fold changes of *IFI27* and *PI3* transcript levels comparing the first day of illness with baseline were also measured by RT-qPCR, and were consistent with the microarray result (S5 Fig).

We also examined the pattern of gene expression in the group of individuals reporting symptoms of acute viral respiratory illness but who were negative in PCR or culture tests for the tested viral pathogens. These individuals had gene expression profiles nearly identical to those observed in the known virus groups, including the acute and recovery phases of gene expression. The transcript levels of *IFI27* and *PI3* in these subjects were more similar to the non-influenza infection cases. Within this group there was some variability in the magnitude of the transcriptional responses, including large variation in *IFI27* and *PI3*, perhaps suggesting either additional etiologic heterogeneity or incomplete sensitivity of the culture and PCR assays (S3 Fig).

Three subjects had a systemic expression profile consistent with activation of interferon signaling on the day of enrollment (Fig 2D). One of these individuals had persistent elevations of these transcripts throughout the study. One subject reporting illness symptoms did not have the signature of acute systemic response on day 0 but had the typical signature by day 4. The remaining 4 subjects showed ‘off-cycle’ activation profiles consistent with additional intercurrent infections with or without severe symptoms.

### Expression changes in lineage and activation state markers reveal increased activated NK cells during the acute phase of influenza infection

**Fig 3:**
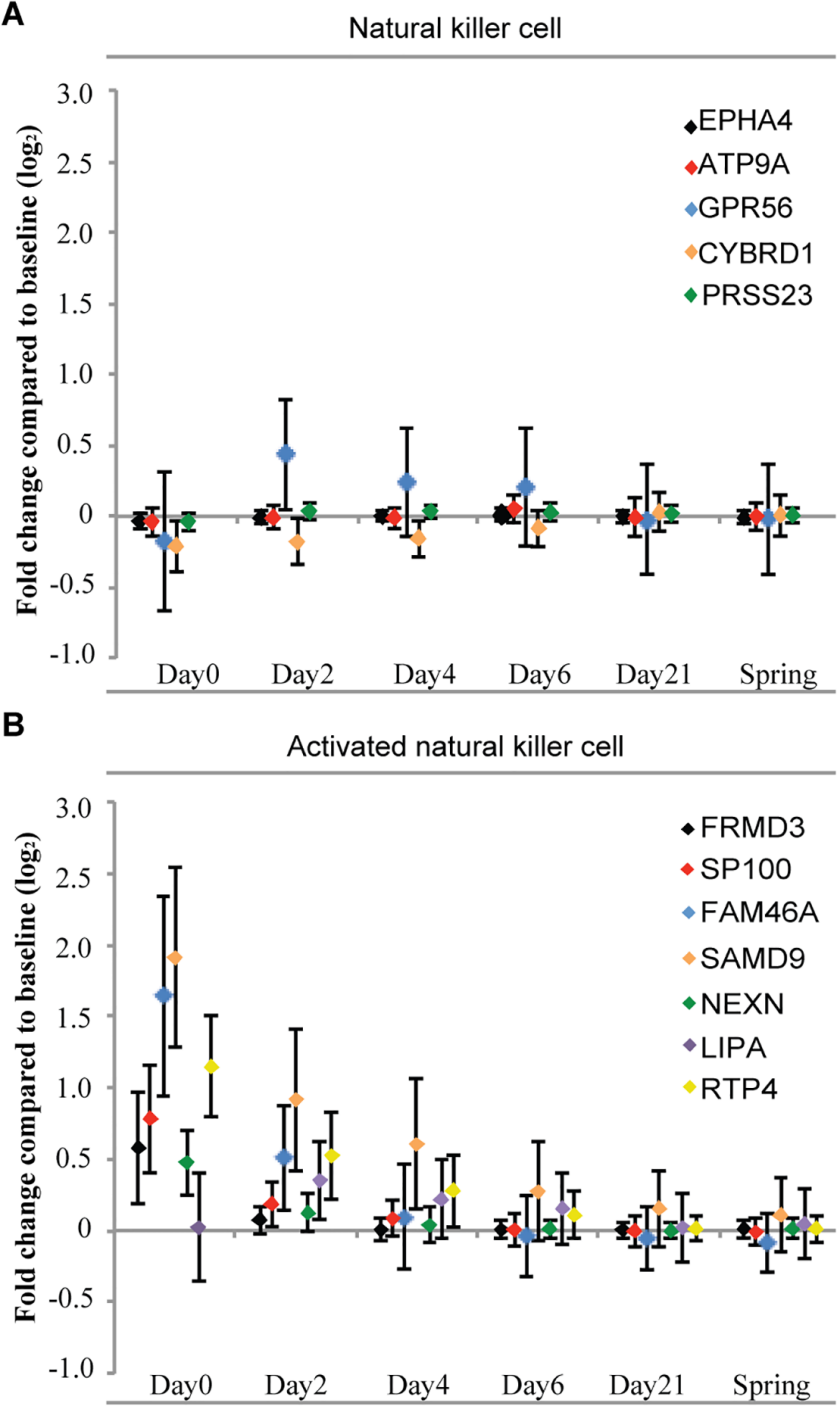
There is little change in the expression of NK cell lineage markers (A), but a significant increase in NK cell lineage activation genes (B) during the course of influenza. Lineage specific transcripts lists were obtained (S3 Table) and then mapped on to the Illumina array probe identifications. The fold changes of the lineage-specific markers on each day represent the differences to the baseline expression levels on a log_2_ scale. Error bars show one standard deviation above and below the average of all the influenza-infected individuals.

We used previously published lineage and activation state marker sets to compute cell type scores for each sample (S3 Table). Lineage specific transcripts lists were obtained and then mapped on to the Illumina array probe identifications. The expression levels of the lineage-specific markers on each day were computed by taking the average of all the influenza-infected individuals. The changes in the expression levels are likely influenced by both the proportion of the cells in the peripheral blood as well as the transcriptional state of those cells. In the acute phase of infection there was a slight depression of lineage markers for NK cells, followed by up-regulation of the marker gene *GPR56* on day 2 that became stable by day 21 and thereafter (Fig 3A). The same procedure was used to compute a score for the activation status. Notably, NK cells showed very dramatic increases in activation state during the acute infection but then the activation signature rapidly resolves in the convalescent phase as the infection subsided (Fig 3B). The changes observed in the 2009 cohort were replicated in the 2010 cohort. Our findings of the dramatic activation of NK cells during the early phase are consistent with the observations from influenza-infected mouse lungs [20]. These data show an intense activation of NK cells during the acute phase of infection.

### Functional annotation and classification of differentially expressed genes induced by ARIs

**Fig 4:**
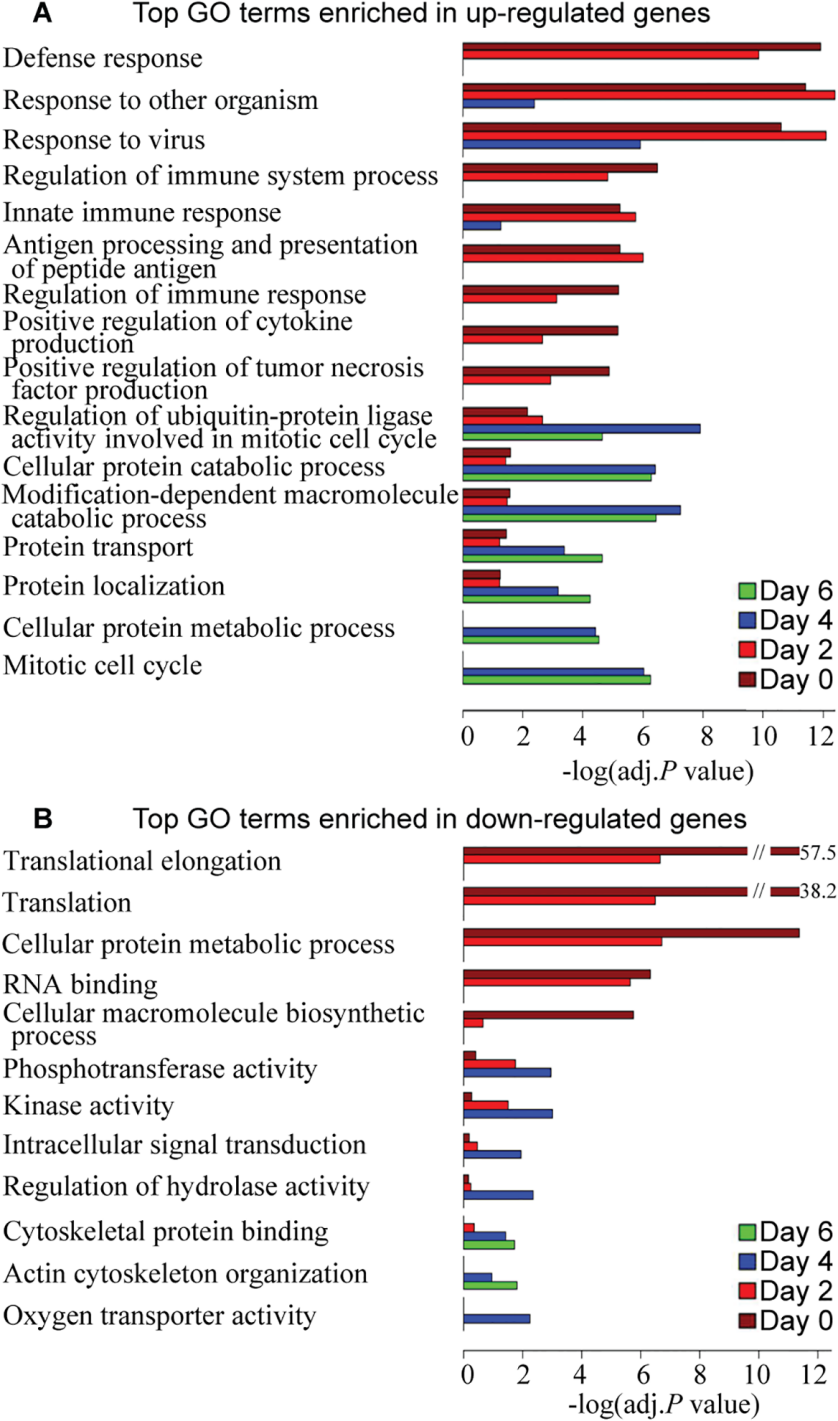
Top GO terms enriched in differentially expressed genes over the course of 6 days after influenza virus infection. DAVID was used to identify over-represented Gene Ontology terms among **(A)** up-regulated genes and **(B)** down-regulated genes on each day (BH-corrected *P* values <0.05 in both 2009 and 2010 cohorts). The length of the bar (x-axis) represents the–log_10_ (Benjamini-adj.*P* value). The bars are colored by day.

After the gene expression status of the peripheral blood cells of influenza-infected individuals were profiled over the time course of illness, we then searched for molecular, cellular and biological processes that best correspond to the host gene expression responses. For this, we analyzed the functional annotation of differentially expressed genes using DAVID gene ontology. Analyses of significant differentially expressed genes on day 0 and day 2 (BH-corrected *P* value <0.05 in both discovery and validation cohorts) revealed that the upregulated genes were mostly enriched in defense response, response to other organism, response to virus, innate immune response, positive regulation of cytokine production, and positive regulation of tumor necrosis factor production (Fig 4A), while the downregulated genes were involved in translational elongation, translation, cellular protein metabolic process, rRNA binding, and cellular macromolecule biosynthetic process (Fig 4B). The functional interpretation of differentially expressed genes in the recovery phase (day 4 and 6) is much less clear: there are a range of protein metabolic process and regulation of ubiquitin-protein ligase activity functions represented in the upregulated genes (Fig 4A), and actin cytoskeleton organization functions associated with the downregulated genes (Fig 4B), but how these changes may be integrated will require further investigation. We observed a higher level of gene enrichment in the interferon signaling and the tumor necrosis factor production pathway in the acute phase of influenza virus infection compared to rhinovirus infection.

### Pathway enrichment analysis and interaction network of differentially expressed genes in acute and recovery phases of the host response to ARIs

We next examined how biological pathways might be altered during the course of influenza with respect to baseline, by performing separate content analyses of DEGs on day 0 or day 6. We found that upregulated genes in acute influenza virus infection were enriched for canonical pathways specific to interferon signaling (S6A Fig), role of pattern recognition receptors of virus, TREM1 signaling, antigen presentation pathway, activation of IRF by cytosolic pattern recognition receptors. Cellular processes such as dendritic cell maturation and crosstalk between dendritic cells and NK cells were also enriched, indicating the activation of these pathways in acute influenza. On the other hand, the downregulated genes were significantly enriched for pathways related to gene translation and cell proliferation, such as EIF2 signaling, regulation of eIF4 and p70S6K signaling and mTOR signaling. A group of pathways distinct from those seen in the acute phase were enriched in DEGs in the recovery phase (S6B Fig). A large number of upregulated genes in the recovery phase were functioning in the protein ubiquitination pathway. Stress response pathways (e.g. hypoxia signaling in the cardiovascular system and NRF2-mediated oxidative stress response) were also enriched. In addition, significant enrichment in multiple growth factor signaling pathways (e.g. GM-CSF signaling, HGF signaling and PDGF signaling) and cell cycle regulation (e.g. mitotic roles of polo-like kinase) were observed.

### Dynamic transcriptional co-expression modules in response to influenza virus infection

Highly co-expressed genes usually share common regulatory mechanisms or participate in the same biological process. To reveal distinct patterns on how host genes are co-expressed in different stages of influenza virus infection, the WGCNA method was applied to the gene expression profiles of samples from the first or second day of illness (day 0), day 2, day 4 and day 6, thereby the network organization is approached through inference of variable gene co-expression patterns and dynamic pathway activity rather than a fixed predefined gene annotations.

**Fig 5:**
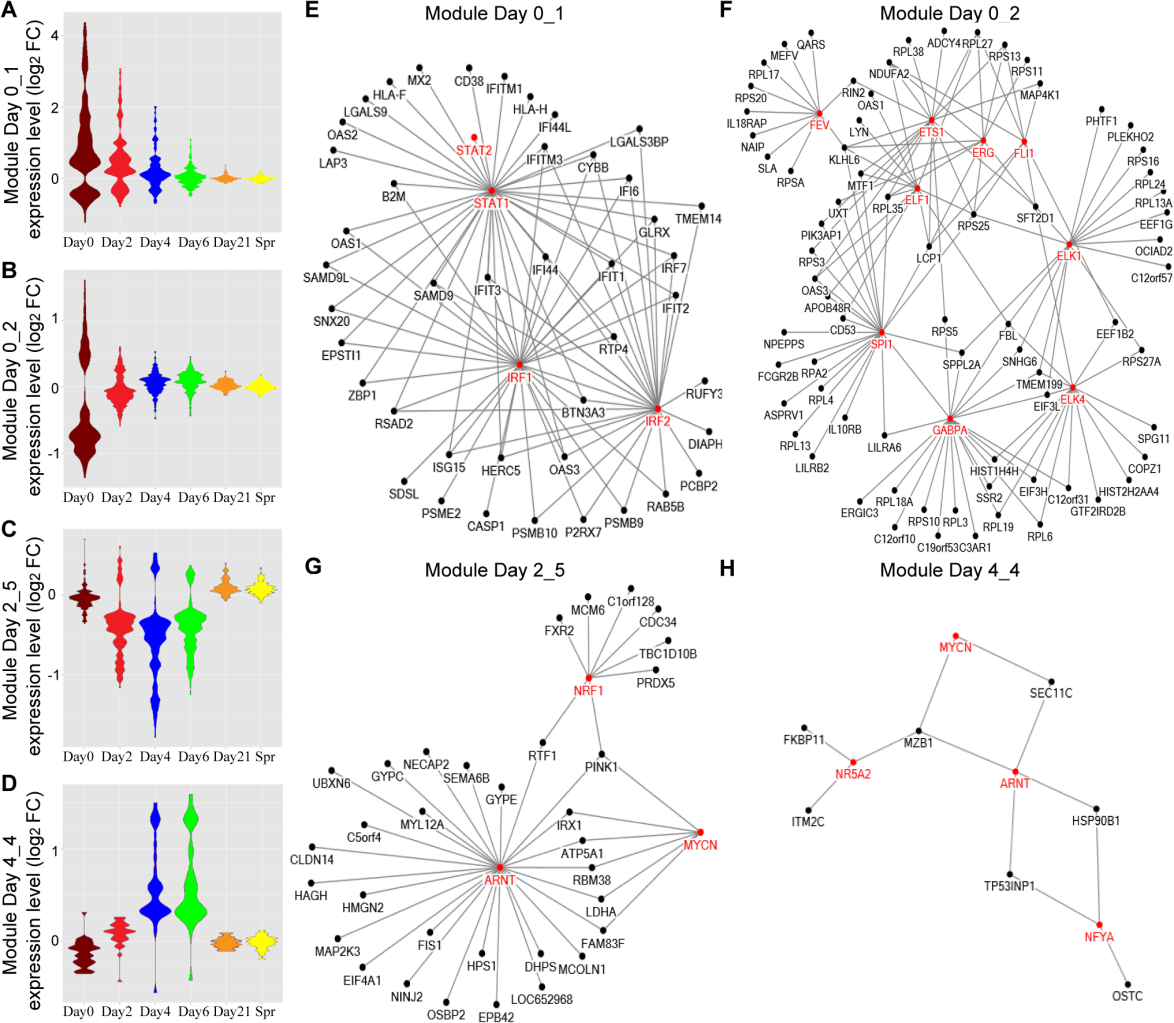
TF networks within the WGCNA modules over the course of influenza illness. **(A-D)** Groups, or modules, of co-regulated DEGs were identified by WGCNA. Representative Gene Ontology (GO) categories for each module were identified by functional enrichment analysis and shown in Table 6. Module expression patterns across different time points were represented by violin plots of log_2_ fold-change in gene expression relative to baseline. **(E-H)** Pscan was used to scan the promoter regions of all genes in each module and identify the over-represented transcription factor binding sites (TFBS). The predicted transcription factors, which marked in red and their target genes (z-score > 2) were connected by edges in the networks.

We examined the differentially expressed transcripts (BH-corrected *P* values <0.05 and |log2 Fold-Change| > 0.3 in both cohorts) and detected 6 co-expression modules on day 0 (designated Day 0_1 to Day 0_6) (Table 6). Module Day 0_1 contains genes that are highly upregulated (Fig 5A), and many of them are interferon signaling pathway genes (e.g. *IFI6*, *IFI44L*, *IFIT1*, *IFIT3*, *IRF7* and *STAT1*). Genes in module Day 0_2 are enriched for translational elongation, the majority of the transcripts in this module are downregulated on day 0 but the transcript levels then gradually increased and became above baseline on day 4 (Fig 5B), suggesting the host translation system was attenuated in the acute phase of influenza but then recovered during the later phase.

**Table 6:** GO functional enrichment analysis for the 26 modules detected by WGCNA on different days after influenza virus infection.

Module	No. of transcripts	Representative GO functional enrichment	Benjamini-adj. *P* value
**Day 0_1**	180	Response to virus	8.8E-4
		Response to other organism	1.2E-2
		Defense response	1.8E-2
**Day 0_2**	281	Translational elongation	1.5E-54
		Cytosolic ribosome	1.5E-42
		Translation	2.5E-38
**Day 0_3**	34	NA	
**Day 0_4**	81	Ribonucleoprotein complex	3.8E-3
**Day 0_5**	419	Response to other organism	1.3E-6
		Response to virus	2.3E-6
		Innate immune response	6.7E-5
**Day 0_6**	128	Translational elongation	3.1E-11
		Cytosolic ribosome	1.1E-9
		Ribosome	3.1E-9
**Day 2_1**	69	Cytosolic ribosome	1.5E-2
		Ribosomal subunit	2.8E-2
**Day 2_2**	428	Response to virus	4.7E-16
		Response to other organism	1.3E-13
		Defense response	7.3E-9
**Day 2_3**	200	Translational elongation	1.2E-17
		Cytosolic ribosome	1.4E-16
		Translation	3.8E-15
**Day 2_4**	47	NA	
**Day 2_5**	169	Hemoglobin complex	7.3E-5
		Oxygen transporter activity	5.3E-3
		Oxygen transport	2.9E-2
**Day 2_6**	111	NA	
**Day 4_1**	103	Generation of precursor metabolites and energy	3.1E-2
**Day 4_2**	60	RNA binding	9.6E-4
**Day 4_3**	120	Receptor activity	6.2E-3
		Protein complex binding	9.4E-3
		Plasma membrane	4.7E-2
**Day 4_4**	32	Endoplasmic reticulum part	1.4E-4
		Nuclear envelope-endoplasmic reticulum network	9.7E-3
		Endoplasmic reticulum membrane	1.1E-2
**Day 4_5**	38	NA	
**Day 4_6**	58	NA	
**Day 4_7**	187	Hemoglobin complex	1.1E-6
		Oxygen transporter activity	1.6E-4
		Oxygen transport	9.4E-4
**Day 4_8**	242	Response to virus	5.9E-16
		Response to other organism	1.5E-12
		Defense response	2.5E-7
**Day 4_9**	45	Caspase inhibitor activity	6.7E-3
		Negative regulation of cell death	1.9E-2
		Negative regulation of programmed cell death	2.7E-2
**Day 6_1**	125	NA	
**Day 6_2**	56	Intracellular organelle lumen	7.7E-3
		Intracellular membrane-bounded organelle	1.1E-2
**Day 6_3**	51	NA	
**Day 6_4**	106	Endoplasmic reticulum membrane	1.4E-2
		Nuclear envelope-endoplasmic reticulum network	1.5E-2
		Endoplasmic reticulum part	2.7E-2
**Day 6_5**	73	NA	

*NA = No significant (Benjamini-adj. *P* value < 0.05) GO term enrichment were observed.

We applied the same method to DEGs identified on day 2, day 4 and day 6 (Table 6). Almost all the GO terms over-represented in day 2 modules were observed on day 0, with the exception of the GO terms enriched in module Day 2_5: Hemoglobin complex / oxygen transport. We found many hemoglobin genes in this module, and their transcript levels were decreased on day 4, including *HBD*, *HBE1*, *HBG1*, *HBG2*, *HBA1*, *etc* (Fig 5C). DEGs on day 4 were grouped into 9 modules and these modules became more diverse in GO term enrichment. A small module Day 4_4 contains some of the top upregulated genes (Fig 5D), such as *IGLL1*, *IGJ*, *LOC652694*, *MZB1*, which are involved in antibody secretion.

To elucidate transcriptional regulatory networks within each module, we performed transcriptional factor (TF) enrichment analysis using Pscan. The results for module Day 0_1, Day 0_2, Day 2_5 and Day 4_4 are shown in the networks (Fig 5E–5H) with the red nodes representing enriched TFs. Module Day 0_1 is regulated by the TFs in the interferon signaling pathway. Module Day 0_2 involves genes targeted by ETS transcription factor family. Meanwhile, the binding motifs of *ARNT* and *MYCN* are enriched in both Module Day 2_5 and Day 4_4. Many genes in these networks contain binding motifs of multiple TFs, implying the TFs are highly coordinated in regulating downstream targets.

### An enhanced gene co-expression pattern after influenza virus infection revealed by differential co-expression analysis

**Fig 6:**
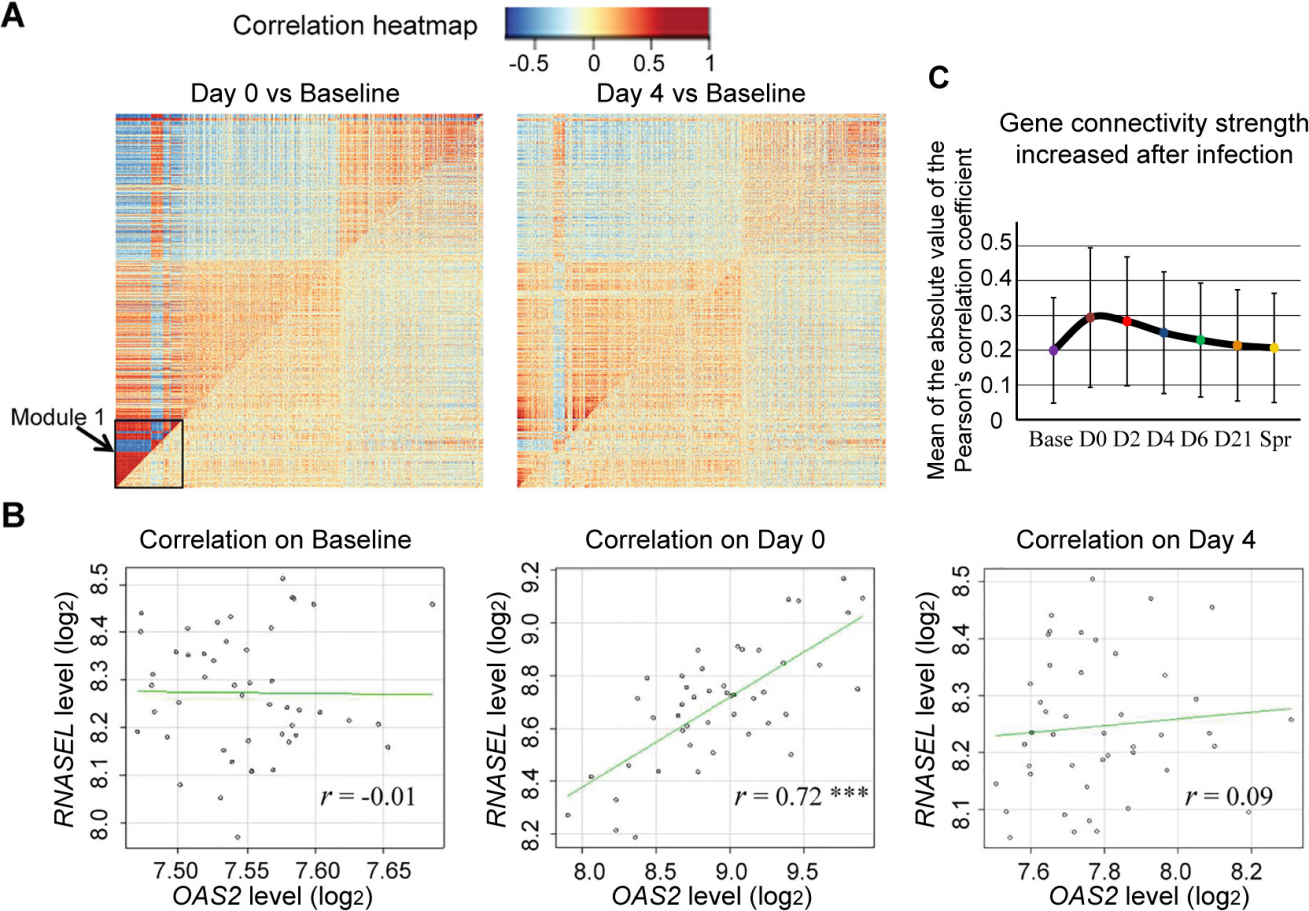
Host gene network connectivity became stronger after the subjects were infected with influenza virus. **(A)** In the comparative correlation heatmap, the upper diagonal of the main matrix shows a correlation between pairs of genes among samples collected from the individuals after influenza virus infection (Left: Day 0, Right: Day 4). The lower diagonal of the heatmap shows a correlation between the same gene pairs in these individuals on baseline. Red color corresponds to positive correlations, and blue corresponds to negative correlations. **(B)** Changes in the correlation between genes *OAS2* and *RNASEL*. Each dot corresponds to an individual and the axes mark the log_2_ expression values of the two transcripts in that individual. The genes are uncorrelated on baseline (*r* = -0.01) but are positively correlated on day 0 (*r* = 0.72, *P* <0.001), and this correlation became attenuated on day 4 (*r* = 0.09).

Differential co-expression patterns, wherein the level of co-expression of gene groups differs between illness and pre-illness, can arise from an influenza infection-related change in the regulatory mechanism governing that set of genes. We performed differential co-expression analysis on all the genes that are differentially expressed on day 0 in influenza-infected individuals. We found that the correlations between gene expression levels of all the gene pairs are higher on day 0 compared to baseline (Fig 6A). Particularly, the modules 1 in the lower left corner have significant correlation differences between day 0 and baseline, suggesting that the module genes are in the same regulatory network. For example (Fig 6B), the correlation coefficient between the expression levels of *OAS2* and *RNASEL* gene are nearly 0 at baseline, yet they became highly correlated (r = 0.72) on day 0. We computed the correlation coefficients among all gene pairs and plotted the results as a function of the magnitude of correlation or connectivity (Fig 6C). The co-expression patterns among these genes peaked on the first day of illness, gradually weakened thereafter, and become indistinguishable with baseline by day 21. The gene expression correlation also increased, though to a lesser extent, in HRV infection group (S7 Fig).

### Genes that show evidence of correlation between gene expression and the magnitude of the antibody response

**Fig 7:**
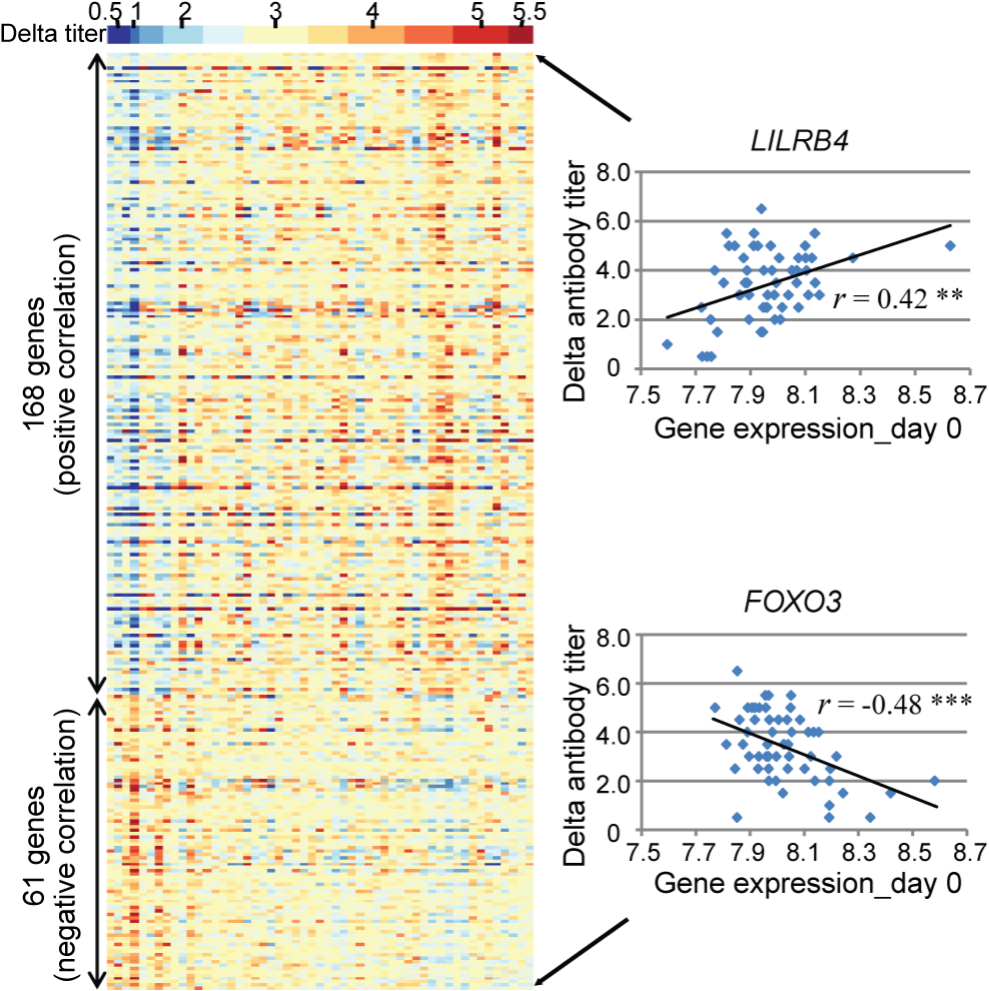
A total of 229 genes showed evidence of significant correlation between gene expression and the antibody response. **(Left)** Each individual is represented by a column in the heatmaps. The top heatmap displays the magnitude of the antibody response (delta titer). The bottom heatmaps display the deviations around the expression mean for each transcript. **(Right)** *LILRB4* showed the greatest positive correlation (*r* = 0.42, *P* <0.005) and *FOXO3* showed the greatest negative correlation (*r* = -0.48, *P* <0.001) between gene expression on day 0 and the magnitude of the antibody response to influenza virus infection.

We measured serum antibody to the pH1N1 viruses in all subjects at enrollment and after surveillance for illness [5, 6], and thus were able to record the magnitude of antibody response (delta H1N1 titers). We wished to identify the genes whose transcripts levels are correlated with antibody response. The Day 0 transcript levels of 2119 DEGs were tested for their correlation with the delta antibody titers among 58 ill subjects with H1N1 infection. We found that a total of 229 genes showed evidence of significant correlation between gene expression on the first day of illness and the antibody response (Fig 7, S4 Table). Of these, 168 showed evidence of positive correlation and 61 of negative correlation. *LILRB4* (Leukocyte immunoglobulin-like receptor subfamily B member 4) showed most significant positive correlation, and a member of the forkhead transcription factors, *FOXO3* exhibited most significant negative correlation. Content analysis revealed that immune response (GO:0006955) were most enriched in the genes showed positive correlation with antibody response (e.g. *OAS1*, *CD14*, *APOBEC3G*, *IFITM3* and *LILRB4*). B-cell proliferation genes (e.g. *CD40*, *SASH3*, *CDKN1A and TICAM1*) were strongly correlated with high antibody response. Genes that showed negative correlation (e.g. *FOXO3*, *DAPK2*, *SGK1*, and *TP53INP1*) were enriched for apoptosis and programmed cell death pathways (GO:0012501).

## Discussion

This prospective study of 2009 pandemic influenza A virus infections and illnesses in healthy adults in a university community detected clear gene expression patterns correlating with moderate influenza. Transcriptional profiles for ill subjects were examined at 7 time points, including baseline, the first day of illness and up to 21 days after as well as after the influenza season. The aim of this study was to use an unbiased genome-wide approach to identify genes whose expression is regulated by occurrence of an ARI and networks that are activated in the host response to infectious stimuli during different stages of an acute respiratory viral illness.

The results show a gene expression signature that strongly corresponds to influenza virus infection. Components of interferon pathway and innate immunity (*IFI44L*, *IFITM3*, *MX1*, *IRF7*, *OAS2*, *STAT2*, *etc*.) are significantly upregulated in the acute phase of infection, while the expression levels of genes involved in translational elongation and protein biosynthesis are decreased. Other researchers have identified host gene expression patterns that are associated with viral infection in human airway epithelial cells [17] and bronchial epithelial cells [28]. DEGs in type I interferon or *STAT1* signaling were similar to those found in our current study. In particular, *IFI27*, an antiviral molecule that regulates interferon-mediated apoptosis, was the most highly up-regulated gene in these studies [14,17]. Our study identified *IFI27* and *PI3* as the most differentially expressed genes comparing influenza virus and rhinovirus infections. *IFI27* up-regulation was observed in hospitalized infants with RSV bronchiolitis [36], yet it was not seen in the three healthy adults infected with RSV in our study. *PI3* (peptidase inhibitor 3) encodes elafin, a potent neutrophil elastase inhibitor, localized to the injury sites in the lung [37]. *PI3* protein was shown to possess antimicrobial and anti-inflammatory activities [38]. Its mechanism of action is, however, poorly understood and down-regulation of *PI3* has been reported previously in patients with acute respiratory distress syndrome (ARDS) [39], but not in studies of viral infections.

A key finding in this study was a recovery phase that involves differential expression of a set of genes distinct from those observed in the acute phase of infection. Molecular characterization of a recovery phase has not been previously reported and the functions in the immune response of most of the differentially expressed genes are much less clear. Protein ubiquitination pathways and protein metabolic process are associated with the genes upregulated in the recovery phase. Symptomatic influenza cases exhibited extensive regulation in multiple growth factor signaling and cell proliferation pathways during the recovery phase. How these results may be integrated will require further investigation.

Comparison of influenza and rhinovirus illnesses indicated that the intensity of the increase in activation of the interferon and innate immunity pathways is less in rhinovirus infections. In addition to *IFI27* and *PI3* that have the largest expression difference between influenza and HRV, *CDKN1A* and *CDKN1C*, which are essential genes involved in cell cycle control, appeared to be differentially expressed specifically in influenza virus infection. It has been reported that influenza virus infection induced cell cycle arrest in G1/S phase [40], and the transcriptional reprogramming of cell cycle correlated with the severity of influenza illness [41]. The differences in host transcriptional response to influenza virus and rhinovirus infection might be explained by the fact that influenza virus replicates in the nucleus of host cells while HRV replicates in cytoplasm [42], or explained by the distinct viral mechanisms in histone modification [22]. Individuals who did not have an identified pathogen associated with illness had conserved systemic expression signatures that were indistinguishable from the influenza and rhinovirus groups, with a large variation in the intensity of transcriptional response. This suggests that they were actually infected with one of the respiratory viruses for which we tested but which was not detected, or that they had an infection with another infectious agent that induces a similar transcriptional response. This question will be the subject of future investigation using highly sensitive next generation sequencing methods.

The finding of ‘out of cycle’ individuals suggests that there are many subclinical infections or other subclinical disorders in healthy adults. Given that acute viral infection stimulates gene pathways known to be involved in adult onset autoimmune disorders raises the possibility that the number and intensity of infections may alter risk in genetically susceptible individuals. One individual exhibited activation at all time points. This may represent a systemic disorder, a possibility that is now being examined in that subject. This suggests the possibility that gene expression profiles may be used in the detection of such disorders as an adjunct to standard immunological testing.

We used weighted gene co-expression network analysis (WGCNA) to cluster the DEGs detected on day 0 –day 6 into 26 modules. This module construction strategy takes advantage of the biological variability inherent in the prospective cohort study in order to uncover the modular organization and function of transcriptional systems. The time-course transcriptional profiles make it possible to study the transcriptional regulation of these gene co-expression networks during different phases of influenza illness. While GO terms enriched in the acute phase modules are “response to virus” and “translational elongation”, recovery phase modules are over-represented in a new set of GO terms, such as “endoplasmic reticulum part”, and “programmed cell death”. We also found the hemoglobin genes in module Day 2_5 (e.g. *HBD*, *HBE1*, *HBG1*) are downregulated in response to influenza virus infection, however, whether this is due to true transcriptional regulation, or a decrease in their percentage in comparison to the white cells will need further investigation. Furthermore, the TF regulatory networks in 4 modules were uncovered, which provides better insights to the underlying mechanisms of host response to ARIs and will facilitate drug and vaccine development.

Our study went beyond gene co-expression and investigated the differential co-expression patterns in influenza virus and rhinovirus infections. The idea behind this is that the identification of changes in gene co-expression patterns between illness and baseline samples could provide information about infection-affected regulatory networks. Our result demonstrates that the gene expression correlations are enhanced on a global scale in the response to ARI; a small module containing 273 transcripts has the largest increase in network connectivity strength. This suggests qualitative change in the gene network upon an infectious stimulus.

We know there are several cell-types in whole blood sample and the proportion of these cell-types varies across samples, so it is possible that the co-expression modules were driven by variation in markers for various cell-types. However, the differences in gene expression correlation between baseline and the first day of illness are so large that it cannot be fully explained by the variation in the expression values of the cell lineage markers, which does not change much between baseline and illness. Thus, the changes in regulatory mechanisms are the major contributor to the differential co-expression patterns.

The pandemic influenza A/H1N1 virus emerged in April 2009 and was the dominant influenza virus circulating in humans in our study periods. By measuring the H1N1 antibody titers on the same individual before and after the influenza season, we were able to record the magnitude of antibody response (delta H1N1 titers) and account for individual variation in a way that would not have been possible otherwise. We identified 229 genes whose transcriptional levels were correlated with the antibody response. Although the sample size we had for the correlation analysis is relatively small, over 1/3 of the genes identified in this study have previously been shown to be correlated with antibody response to influenza vaccination [24]. B cell proliferation genes, which predict influenza vaccine-induced antibody response [23], were also correlated with the antibody response to naturally occurring influenza infection. These findings provide more insight into the molecular mechanisms of antibody production and secretion, and may also contribute to influenza vaccine development.

Several limitations of this study are noteworthy. First, we studied two cohorts of healthy young adults. Those subjects who subsequently developed influenza-like illness had moderate symptoms [5]. Children, the elderly, were not included and, fortunately, none of the research subjects developed severe illnesses. Second, this study did not allow analysis of the subjects who had influenza infection without symptoms. Based on seroconversion rates, 38% of the subjects were probably infected with influenza A H1N1 but did not have symptoms sufficient to trigger a follow up study visit. Third, all the subjects with influenza-illness enrolled in year 2009–2010 were infected with influenza A H1N1. And in year 2010–2011, only 9 were infected with influenza B and 3 were infected with influenza A H3N2, all the others were infected with H1N1 infection. Thus the sample size was not sufficient for comparing host transcriptional response to influenza A H1N1, H3N2 and influenza B. Fourth, the transcriptional responses to infection of cells residing in the secondary immune tissues, like lymph nodes or spleen, might be different from that of peripheral blood. Future research may investigate the correlations of gene expression between cells residing in different tissues. Finally, while antibody titers have been used to assess humoral immune responses, it is clear that they do not capture the complexity of the host response to ARIs. Additional studies would be necessary to establish the causal relationship between the genes identified and the antibody response, and whether they also regulate cytokine or chemokine levels.

Despite these limitations, the findings in this study demonstrate the power of serial measurements of gene expression, within the context of a prospective clinical trial, to identify candidate genetic mechanisms that determine responses to infection. We have genotyped all these research subjects and have begun analyzing the impact of common genetic variation on the gene expression patterns. Because we have made repeated measurements on the same individual over time, we should be able to account for the effect of person in a way that would not have been possible using cross-sectional methodologies. The dynamic nature of the measurements should also allow the identification of genetic effects that are either enhanced by or only evident after the strong perturbation of acute infection.

## Materials and Methods

### Ethics statement

The study was conducted at Texas A&M University, College Station, TX. The protocol and informed consent were approved by the Baylor College of Medicine and Texas A&M University institutional review boards before the study began. Healthy adults age 18 to 49 at the college and in the community were invited to enroll to be followed for acute respiratory illness (ARI) through two consecutive influenza seasons 2009–2010 and 2010–2011. All adult subjects provided written informed consent.

### Human subjects

After subjects provided consent, a medical history was taken to ensure good health, and baseline specimens were obtained. Surveillance for influenza began during the September 2009 enrollment period because pH1N1 as a cause of influenza was identified in the population during enrollment. Subjects were given thermometers and instructions to call and report for evaluation within 48 hours of onset for any ARI (Fig 1A). Except for the Thanksgiving holiday period and 4 weeks of the Christmas holiday period, a coordinator and physician enrolled persons presenting within 48 hours of onset with a new ARI with fever or that caused them to miss school, work, or social activities. Specimens were obtained and medical care was provided, including the antiviral zanamivir if indicated. Enrolled persons were seen 2, 4, and 6 days later for repeat evaluation, specimen collections, and medical care and 21 days later for collection of convalescent specimens. These subjects are those included in the present report. Surveillance for influenza was terminated after 5.5 months; all subjects were asked to return for specimen collection and to provide a medical and ARI history. The study was repeated 2010–2011 with surveillance for influenza limited to January to April as community surveillance did not detect influenza before the Christmas break.

### Illnesses

A study physician obtained an oral temperature, completed a symptom survey, and performed a respiratory system examination at each illness visit. All cases were classified as clinically moderate using standard criteria.

### Serology

Serum specimens obtained at enrollment, acute and convalescent visits for illnesses, and the terminal visit were tested simultaneously using hemagglutination-inhibition (HAI) antibody tests following previously described methods. Virus antigens were a locally obtained pH1N1 virus (A/Baylor/09) and the most recently prevalent seasonal A/H1N1 virus (A/Brisbane/59/07), A/H3N2 virus (A/Perth/16/09), and B virus (B/Brisbane/60/08).

### Virus infections

A combined 8-mL nasal wash and throat swab specimen was collected at each illness visit. Specimens from the day 0 and 2 visits were tested for all respiratory viruses in tissue cultures. All specimens were also tested by reverse-transcriptase polymerase chain reaction (RT-PCR) for respiratory viruses including influenza A, pH1N1 influenza, influenza B, picornavirus/rhinovirus, respiratory syncytial virus, human metapneumovirus, parainfluenza viruses, coronaviruses, and adenoviruses.

### RNA purification from peripheral whole blood

We collected peripheral whole blood samples (2.5 mL) in PAXgene RNA stabilization tubes (QIAGEN Inc., Valencia, CA, U.S.A.) at each visit of those enrolled for illness and froze the samples at—80°C until RNA purification to minimize gene expression changes induced by handling and processing. RNA purification was performed using the PAXgene Blood RNA system (QIAGEN Inc., Valencia, CA) according to manufacturer’s instructions. Quality control of RNA samples was performed using spectrophotometry (NanoDrop-1000 Spectrophotometer, Thermo Fisher Scientific, Waltham, MA, U.S.A.) and microfluidic electrophoresis (Experion Automated Electrophoresis System, Bio-Rad Laboratories, Hercules, CA).

### Gene expression profiling

#### cRNA synthesis

We performed *in vitro* transcription assay on all peripheral whole blood RNA samples prior to chip hybridization. Ambion Illumina TotalPrep RNA Amplification Kits for 24 reactions (Applied Biosystems/Ambion, Austin, TX) were used according to the manufacturer’s protocol. Briefly, 11uL of 50ng/uL total RNA was reverse-transcribed with an oligo(dT) primer bearing a T7 promoter, using ArrayScript reverse transcriptase (RT). The cDNA then went through second strand synthesis to produce the template for in vitro transcription with T7 RNA Polymerase. MEGAscript in vitro transcription (IVT) and biotin UTP (provided in the kit), were used to generate biotinylated, antisense RNA copies (cRNA) of each mRNA in a sample from the template cDNA. cRNA samples were eluted once using 40 uL nuclease-free water. Spectrophotometry (NanoDrop-1000 Spectrophotometer, Thermo Fisher Scientific, Waltham, MA, U.S.A.) and microfluidic electrophoresis (Experion Automated Electrophoresis System, Bio-Rad Laboratories, Hercules, CA) were performed to QC samples before hybridization onto Illumina Human HT-12v4 Expression BeadChips (Illumina, San Diego, CA).

#### Illumina Expression BeadChip Assay

Biotin-labeled RNA (cRNA) was hybridized to Illumina Human HT-12v4 Expression BeadChips (Illumina, San Diego, CA) according to the manufacturer’s protocol and scanned on an Illumina iScan Reader (Illumina, San Diego, CA). The initial standard quality control thresholds were imposed on the raw intensity signals of the microarray using the iScan Control Software (Illumina, San Diego, CA). Microarrays which failed this QC step were excluded from the analysis.

#### Microarray data processing

Initial quality control of the signal intensity data was performed on the transcript probes in the microarray using the algorithms in the *lumi* package in R statistical software, version 2.14.1 [43]. Integration of raw probe profiles was carried in GenomeStudio software (Illumina). Background adjustment, variance stabilization transformation, and rank invariant normalization were carried out using corresponding functions in the R package lumi. A detection *p*-value cut-off of 0.05 was imposed on the normalized intensities to consider a transcript as detected. All differential expression analyses were restricted to 17,708 transcript probes in the microarray with detection *P* values ≤ 0.05 in at least 70% of the samples.

### Differential gene expression analyses

All statistical analyses on the gene expression data were performed in R Statistical Software [44], version 2.14.1. Differential gene expression analyses with cell composition covariates contrasting the individual day-specific data with the baseline sample obtained at the time of enrollment were performed using function for linear model fitting in the *limma* R package [45]. The significance of differences in gene expression was tested using a Bayes moderated t-test [46]. Correction for multiple testing was addressed by controlling the false discovery rate (FDR) using the Benjamini and Hochberg (B.H.) method. A transcript probe was considered significantly differentially expressed if the B.H. corrected *P* value was < 0.05. The heatmap function in R Statistical Software was used to generate a heatmap of mean-centered normalized expression values.

### Cell decomposition analysis

Gene expression profiles were investigated for correlation with cell composition in the whole blood. Cell lineage and activation state markers were used as described in Abbas et al [47]. A full list of marker genes we used to compute the cell scores is provided in **S1 Table.** Cell lineage scores for all individuals were obtained by taking the first Principal Component (PC1) of average-normalized expression values for each of the lineage-specific gene sets. [When this method was used to compute the cell scores for the 121 subjects whose whole blood transcriptional profile and Complete Blood Count (CBC) are publicly available at Gene Expression Omnibus (GEO accession: GSE30119), the resulting expression-based lymphocyte and neutrophil scores showed a high correlation (r^2^ = 0.64 and 0.65, respectively) with actual measurements of percent lymphocyte and neutrophil in the CBC (S8 Fig).] Neutrophil and lymphocyte scores were then introduced as quantitative covariates in the linear models of the differential expression analyses to account for the differences in cellular composition between individuals.

### Real-time PCR

TaqMan (Applied Biosystems, Foster City, CA) quantitative real-time reverse transcriptase polymerase chain reaction (RT-PCR) was performed on baseline and Day 0 paired RNA samples from 18 randomly selected subjects with influenza infection only and 11 subjects with rhinovirus infection only. cDNA was first synthesized from approximately 2 ug of total RNA in a 20-ul reaction volume using the High Capacity RNA to cDNA kit (Applied Biosystems). TaqMan probes, available as “Assay on Demand”, were used in the analyses of the expression levels of 2 target genes, *IFI27* (Hs01086373_g1) and *PI3* (Hs00160066_m1), as well as endogenous control gene *GAPDH* (Hs03929097_g1). Quantitative RT-PCR was performed on 1ul of cDNA in triplicates with the CFX96 Touch Real-Time PCR Detection System (Bio-Rad, Hercules, CA). The fold increase in mRNA expression was determined using the ΔΔC_T_ method with the baseline sample of each pair as calibrators.

### Functional enrichment and pathway analysis

Gene lists were analyzed using Ingenuity Pathway Analysis (IPA) software and DAVID Ontology (http://www.david.abcc.ncifcrf.gov) to identify significantly enriched pathways. Expressed genes represented in the full dataset were used as the background. The Biological Process, Molecular Function and Cellular Component subsets of the Gene Ontology (GO) were used for enrichment analysis. DAVID Ontology uses t-test to derive *P* values and applies the Benjamini-Hochberg method to correct for multiple testing. IPA uses a right-tailed Fisher’s exact test to derive *P* values for identifying significantly overrepresented pathways. A smaller *P* value indicates the overrepresentation of a pathway or a GO term by the DEGs is less likely due to random chance.

### De-novo network (module) analysis

To identify groups of host transcripts that showed coordinated regulation in response to acute illness, we applied the weighted gene co-expression network analysis (WGCNA) [29]. The WGCNA method constructs networks or modules consisting of groups of genes that are highly correlated across a set of samples. Briefly, the absolute value of the Pearson correlation coefficient is calculated for all pairwise comparisons of gene-expression values. The Pearson correlation matrix is then weighted and transformed into an adjacency matrix. WGCNA uses the topological overlap matrix based dissimilarity measure as input of hierarchical clustering. A dendrogram (cluster tree) of the network is then obtained from hierarchical clustering. Finally, modules are defined by cutting branches off the dendrogram. WGCNA was performed using the WGCNA package provided in R software.

### Transcription factor binding site motifs enrichment analysis

JASPAR is an open-access database (http://jaspar.cgb.ki.se) derived exclusively from sets of nucleotide sequences experimentally demonstrated to bind transcription factors. Transcription factor binding specificity is represented by position-specific scoring matrices (PSSM) in JASPAR. Employing the profiles available in JASPAR, Pscan (http://www.beaconlab.it/pscan) scans a set of sequences (promoters positions -450 to +50 with respect to the transcription start site) from co-regulated or co-expressed genes and identifies the enriched transcription factor binding site motifs by comparing the average matching value of the matrix on the sequences analyzed and that on the whole promoter set (same set of regions with respect to the transcription start site) of the same organism. Z-test was used to derive *P* values and Bonferroni method was applied to correct for multiple testing.

### Differential co-expression analysis

All the transcripts showed differential expression on Day 0 were used in the differential co-expression analysis (DiffCoExpr) [31]. To detect changes in correlations between gene pairs within module and also between pairs of modules during ARIs, DiffCoExpr, an untargeted approach in which gene modules are not pre-defined, was carried out for each day contrasted with the baseline. Briefly, an adjacency matrix as the Spearman correlation coefficients for all pairs of genes was built for each day and baseline. Then, the correlation changes on each day compared to baseline were quantified by the difference between signed squared correlation coefficients. Finally, the Topological Overlap based dissimilarity matrix was derived from the adjacency change matrix, and was used as input for gene clustering and module detection.

## Supporting Information

S1 Fig Dynamics of global gene expression changes after influenza virus infection. Differential expression analysis was performed for each day, contrasted to baseline. **(A)** A total of 4,706 differentially expressed genes (BH-corrected *P* values <0.05 in both 2009 and 2010 cohorts) were identified over the course of 6 days after influenza virus infection, **(B)** 1140 of the DEGs also passed the threshold |log_2_ Fold-Change| > 0.3. Bars indicate the number of DE genes on each day. Colors indicate the day on which differential expression of the genes were newly detected, e.g. brown: differentially expressed genes newly detected on day 0 compared to baseline; blue: differentially expressed genes that appeared at day 4 and were not differentially expressed at any time before.
(TIF)

S2 Fig The magnitude of the transcriptional response varied between individuals in (A) influenza virus infections and (B) rhinovirus infections. Fold change of the acute phase genes and recovery phase genes were computed in each individual comparing each illness day and baseline. A full list of the genes is provided in S1 Table. Each dot represents the average |log_2_ FC| of all the acute phase genes or recovery phase genes in a subject after infection. White background indicates 2009 cohort and grey background indicates 2010 cohort.
(TIF)

S3 Fig Host transcriptional response to non-influenza virus infections involved the same transcripts that were differentially expressed in the influenza virus infection. Heatmap was plotted as in Fig 3 with the identical transcript list. Subject were grouped by infections status as represented by different colors above columns–Orange = Entero+HRV, Yellow = Entero, Grey = HKU1+HRV, White = HKU1, Purple = NL63+HRV, Light Blue = NL63, Brown = RSV+HRV, Gold = RSV, Black = Unknown. Five individuals with FluA infection and five with HRV infection were included in the heatmap for comparison purposes (Red = FluA, Green = HRV).
(TIF)

S4 Fig Heatmaps demonstrating the time course of the genes showing the most significant pattern of differential expression comparing influenza virus with rhinovirus infection. (A) 2009 Cohort, (B) 2010 Cohort. Each column corresponds to an individual RNA sample and each row represents the mean-centered, normalized expression values for each of the differentially expressed genes (BH-corrected *P* values <0.0001). Samples were grouped by day and subjects were grouped by infections status (influenza virus infection group includes influenza A, influenza B, influenza A +rhinovirus and influenza B +rhinovirus infections). The transcripts fall into 3 groups: 1. transcripts that had contrasting fold-changes between influenza virus and rhinovirus infection group; 2. transcripts that were responsive to rhinovirus infection but had no change in influenza virus infection; 3. transcripts that were responsive to influenza infection but had no change in rhinovirus infection. A full list of the transcript probes in the heatmaps and their corresponding genes is provided in S2 Table. **(C)** *CDKN1A*, *CDKN1B* and *CDKN1C* are among the DEGs detected when comparing influenza virus and rhinovirus infection. Fold Changes of *CDKN1A*, *CDKN1B* and *CDKN1C* were measured in paired day 0 –baseline samples.
(TIF)

S5 Fig The expression changes of *IFI27* and *PI3* measured by RT-PCR are consistent with the microarray data. Fold Changes of *IFI27* and *PI3* transcript levels were measured in paired day 0 –baseline samples by microarray (Black) and qPCR (Grey). Subjects are grouped by infections status—Left = FluA (N = 14), Middle = FluB (N = 4), Right = HRV (N = 11).
(TIF)

S6 Fig Canonical pathways enriched by differentially expressed genes on (A) day 0 and (B) day 6 after influenza virus infection, as determined by Ingenuity Pathway Analysis (http://www.ingenuity.com). The percentage indicates the proportion of upregulated (red) and downregulated (green) genes in relative to all the genes present in a pathway. The numbers at the end of columns indicate the total number of genes in that pathway. The–log (*p-value*) increases as a pathway is more significantly associated (as indicated by the orange dot along the x-axis).
(TIF)

S7 Fig Gene network connectivity became stronger after the subjects infected with rhinovirus. In the comparative correlation heatmap, the upper diagonal of the main matrix shows a correlation between pairs of genes among samples collected from HRV-infected individuals on the first day of illness. The lower diagonal of the heatmap shows a correlation between the same gene pairs in these individuals on baseline. Red color corresponds to positive correlations, and blue corresponds to negative correlations.
(TIF)

S8 Fig PC1 of lymphocyte and neutrophil markers are highly correlated with actual measurement of cell proportions for the 121 individuals in GSE30119. The first principle components (PC1) of average-normalized expression values of lymphocyte, neutrophil and monocyte specific genes (See S3 Table for the list of lineage specific genes) were plotted along the x-axis. Percent lymphocyte, neutrophil and monocyte in the blood were transformed by quantile normalization and plotted along the y-axis. The squared correlation coefficients (r^2^) between PC1 and cell proportions for lymphocyte, neutrophil and monocyte were 0.64, 0.65, and 0.16 respectively. Liner regression line is shown in red and the black lines represent 95% prediction interval.
(TIF)

S1 Table A list of the transcript probes that were plotted in the time-course expression heatmaps contrasting aris and baseline (enrollment). (DOCX)

S2 Table A list of the transcript probes that were plotted in the time-course expression heatmaps contrasting influenza and rhinovirus infections. (DOCX)

S3 Table Cell lineage and activation state markers selected from the published literatures for computing the cell scores. (DOCX)

S4 Table Genes showed evidence of correlation between gene expression on day 0 and the magnitude of antibody response. (DOCX)

S5 Table Differentially expressed genes on day 0 – day 6 of influenza virus infection. (XLSX)
