# Supplementary figures and images for: Host Transcriptional Response to Influenza and Other Acute Respiratory Viral Infections – A Prospective Cohort Study
Source: PLoS Pathog. 2015 Jun 12;11(6):e1004869. doi: 10.1371/journal.ppat.1004869 (PMC4466531; doi:10.1371/journal.ppat.1004869)

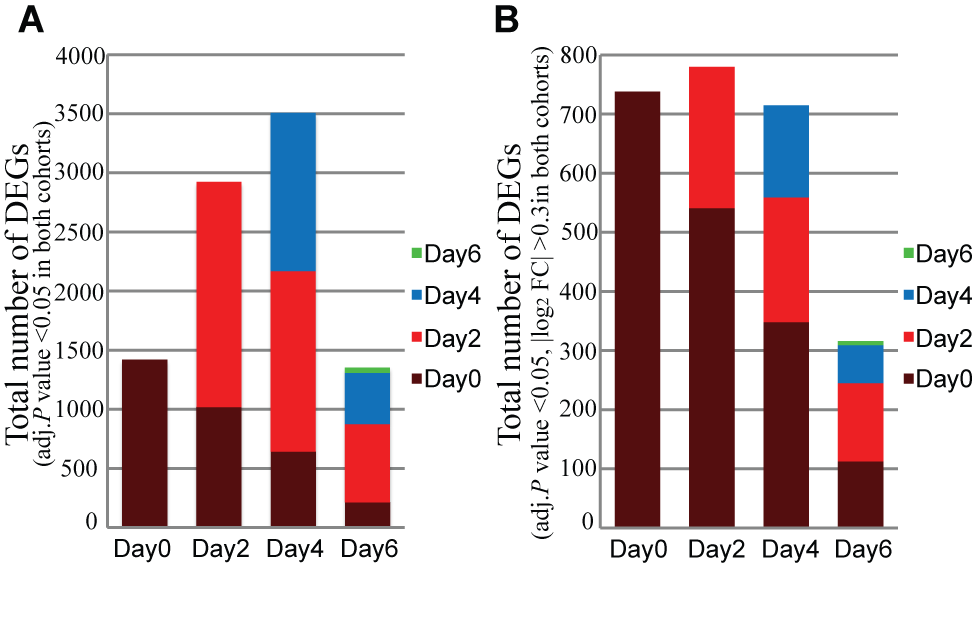

Supplement: S1 Fig — Differential expression analysis was performed for each day, contrasted to baseline. (A) A total of 4,706 differentially expressed genes (BH-corrected P values <0.05 in both 2009 and 2010 cohorts) were identified over the course of 6 days after influenza virus infection, (B) 1140 of the DEGs also passed the threshold |log2 Fold-Change| > 0.3. Bars indicate the number of DE genes on each day. Colors indicate the day on which differential expression of the genes were newly detected, e.g. brown: differentially expressed genes newly detected on day 0 compared to baseline; blue: differentially expressed genes that appeared at day 4 and were not differentially expressed at any time before. (TIF) [file ppat.1004869.s001.tif]

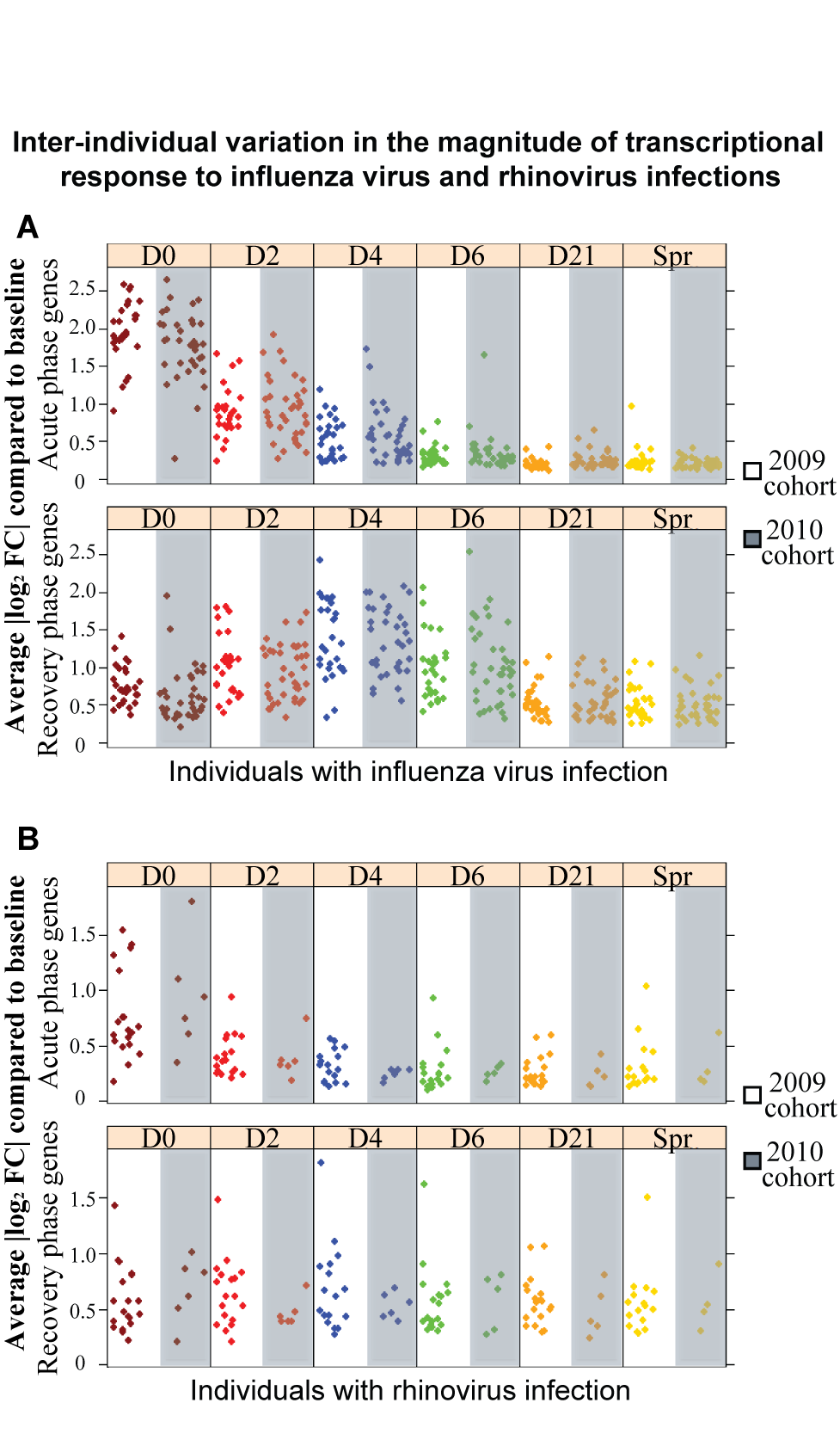

Supplement: S2 Fig — Fold change of the acute phase genes and recovery phase genes were computed in each individual comparing each illness day and baseline. A full list of the genes is provided in S1 Table. Each dot represents the average |log2 FC| of all the acute phase genes or recovery phase genes in a subject after infection. White background indicates 2009 cohort and grey background indicates 2010 cohort. (TIF) [file ppat.1004869.s002.tif]

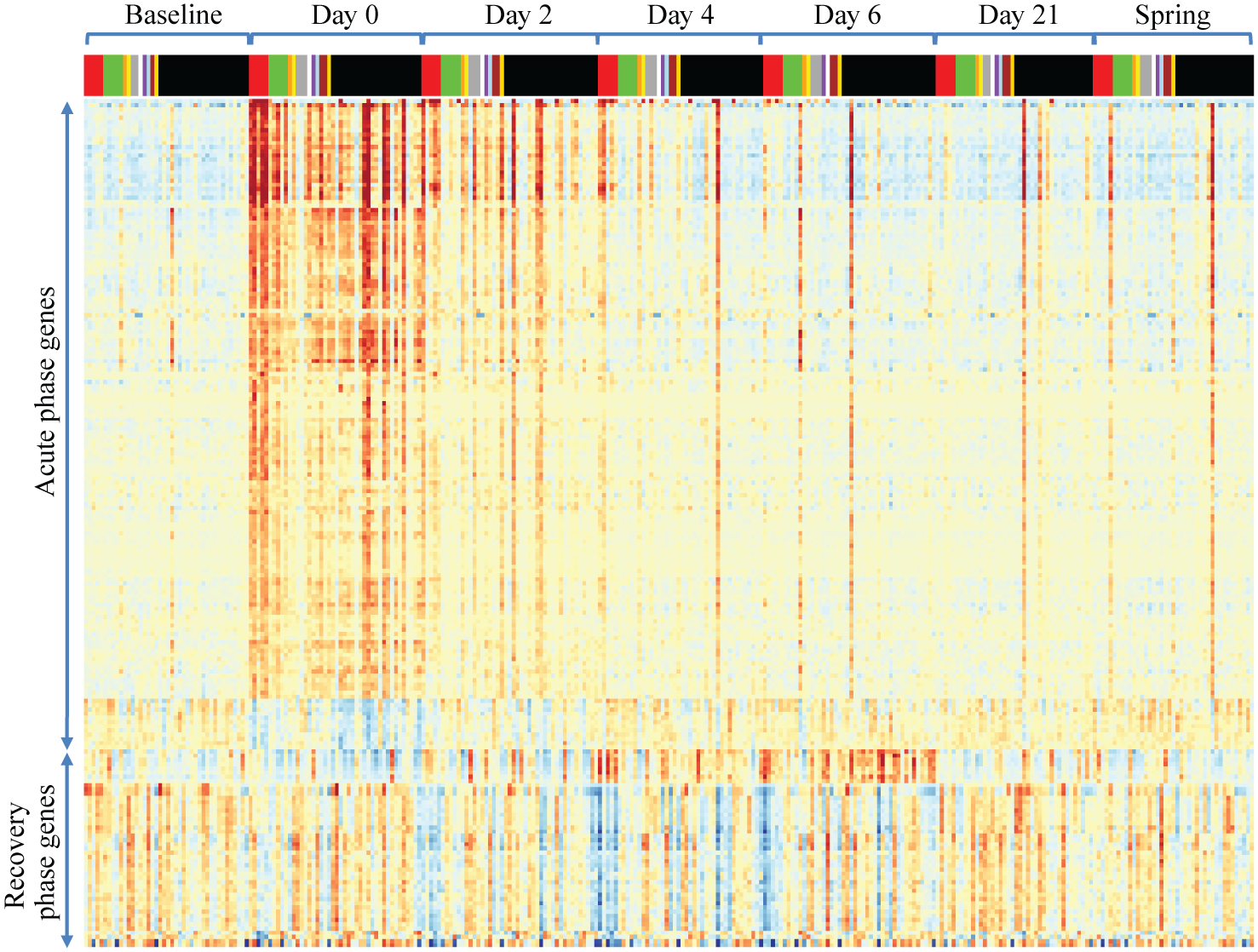

Supplement: S3 Fig — Heatmap was plotted as in Fig 3 with the identical transcript list. Subject were grouped by infections status as represented by different colors above columns–Orange = Entero+HRV, Yellow = Entero, Grey = HKU1+HRV, White = HKU1, Purple = NL63+HRV, Light Blue = NL63, Brown = RSV+HRV, Gold = RSV, Black = Unknown. Five individuals with FluA infection and five with HRV infection were included in the heatmap for comparison purposes (Red = FluA, Green = HRV). (TIF) [file ppat.1004869.s003.tif]

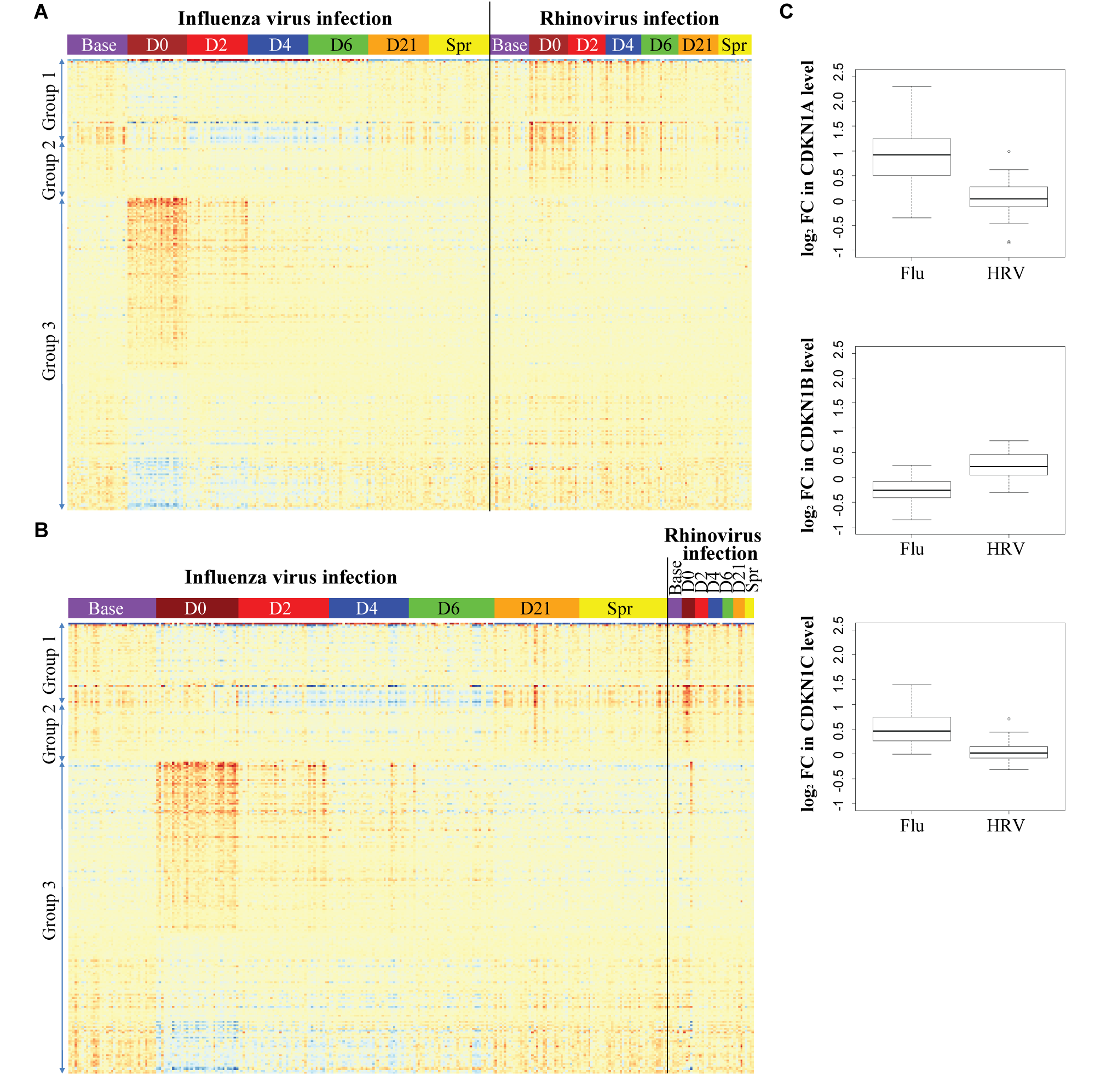

Supplement: S4 Fig — (A) 2009 Cohort, (B) 2010 Cohort. Each column corresponds to an individual RNA sample and each row represents the mean-centered, normalized expression values for each of the differentially expressed genes (BH-corrected P values <0.0001). Samples were grouped by day and subjects were grouped by infections status (influenza virus infection group includes influenza A, influenza B, influenza A +rhinovirus and influenza B +rhinovirus infections). The transcripts fall into 3 groups: 1. transcripts that had contrasting fold-changes between influenza virus and rhinovirus infection group; 2. transcripts that were responsive to rhinovirus infection but had no change in influenza virus infection; 3. transcripts that were responsive to influenza infection but had no change in rhinovirus infection. A full list of the transcript probes in the heatmaps and their corresponding genes is provided in S2 Table. (C) CDKN1A, CDKN1B and CDKN1C are among the DEGs detected when comparing influenza virus and rhinovirus infection. Fold Changes of CDKN1A, CDKN1B and CDKN1C were measured in paired day 0 –baseline samples. (TIF) [file ppat.1004869.s004.tif]

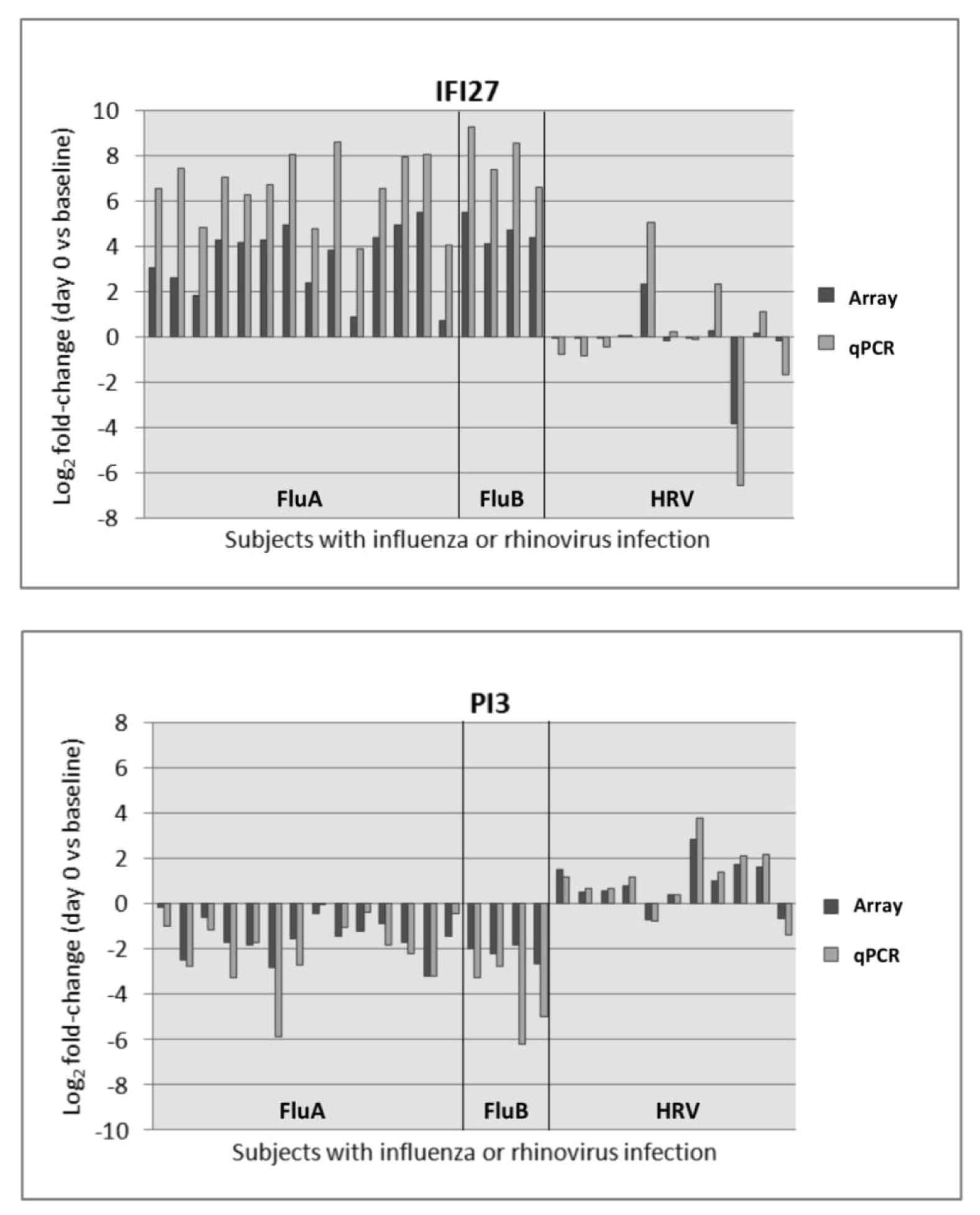

Supplement: S5 Fig — Fold Changes of IFI27 and PI3 transcript levels were measured in paired day 0 –baseline samples by microarray (Black) and qPCR (Grey). Subjects are grouped by infections status—Left = FluA (N = 14), Middle = FluB (N = 4), Right = HRV (N = 11). (TIF) [file ppat.1004869.s005.tif]

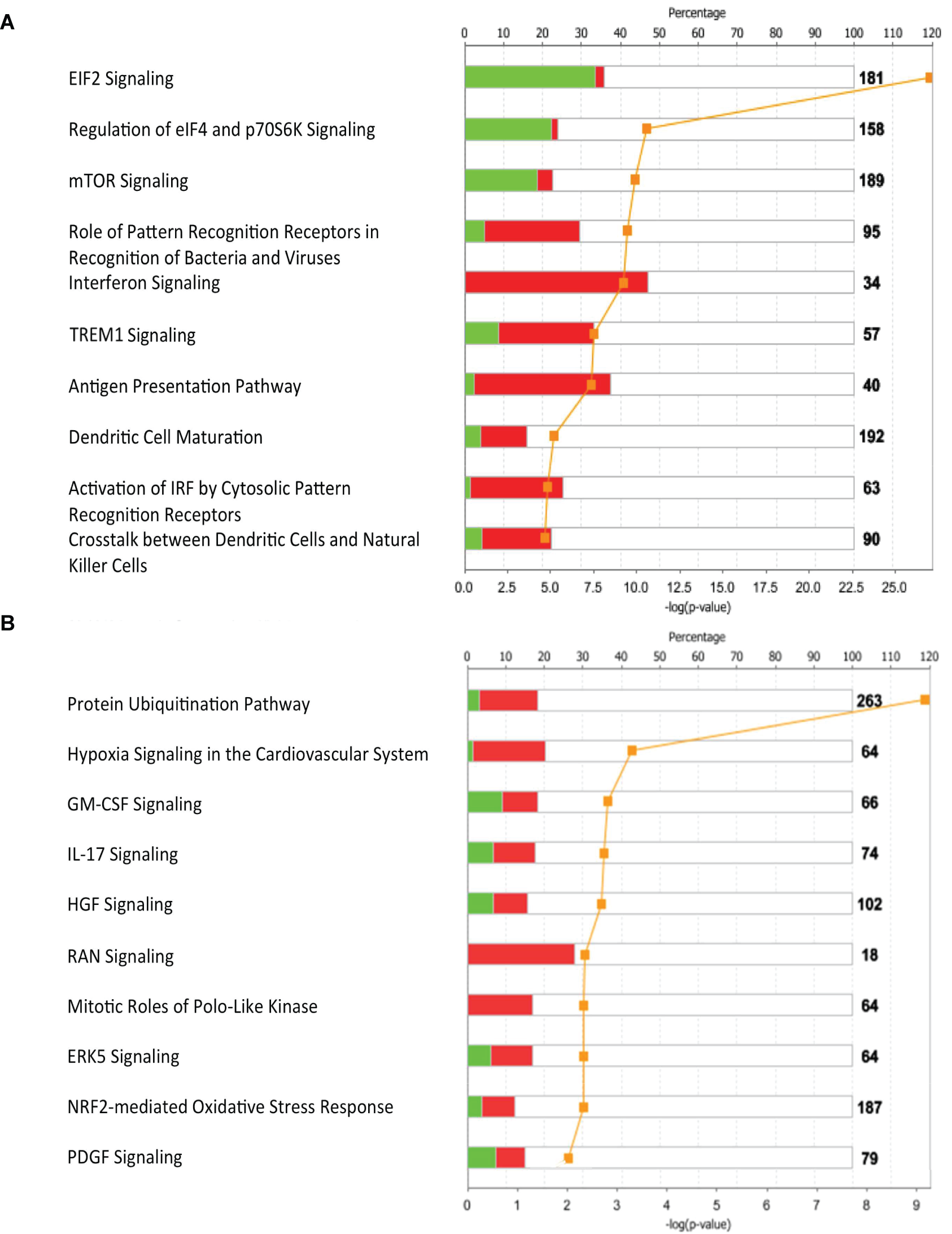

Supplement: S6 Fig — The percentage indicates the proportion of upregulated (red) and downregulated (green) genes in relative to all the genes present in a pathway. The numbers at the end of columns indicate the total number of genes in that pathway. The–log (p-value) increases as a pathway is more significantly associated (as indicated by the orange dot along the x-axis). (TIF) [file ppat.1004869.s006.tif]

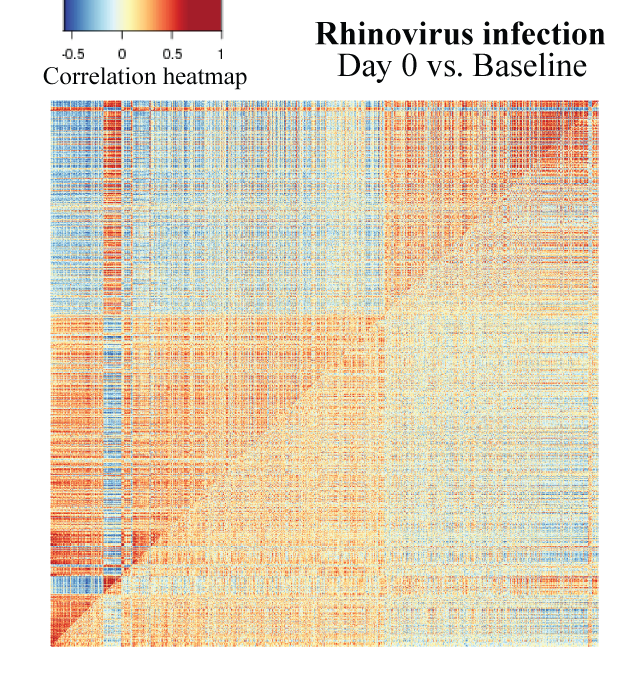

Supplement: S7 Fig — In the comparative correlation heatmap, the upper diagonal of the main matrix shows a correlation between pairs of genes among samples collected from HRV-infected individuals on the first day of illness. The lower diagonal of the heatmap shows a correlation between the same gene pairs in these individuals on baseline. Red color corresponds to positive correlations, and blue corresponds to negative correlations. (TIF) [file ppat.1004869.s007.tif]

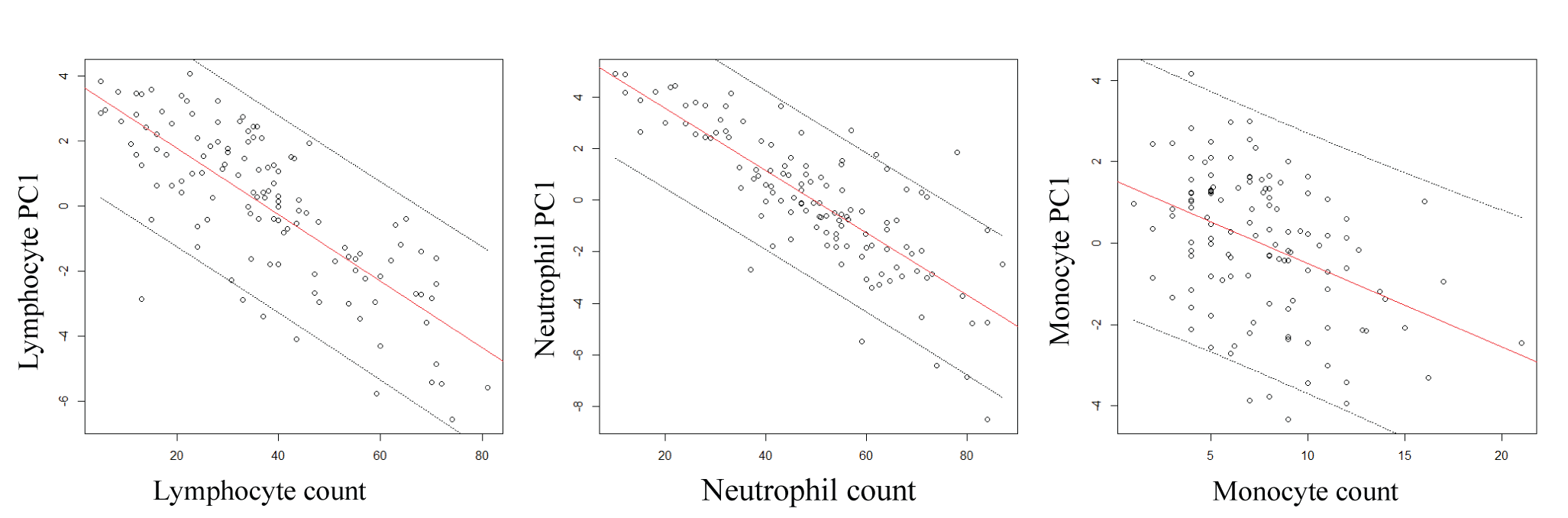

Supplement: S8 Fig — The first principle components (PC1) of average-normalized expression values of lymphocyte, neutrophil and monocyte specific genes (See S3 Table for the list of lineage specific genes) were plotted along the x-axis. Percent lymphocyte, neutrophil and monocyte in the blood were transformed by quantile normalization and plotted along the y-axis. The squared correlation coefficients (r2) between PC1 and cell proportions for lymphocyte, neutrophil and monocyte were 0.64, 0.65, and 0.16 respectively. Liner regression line is shown in red and the black lines represent 95% prediction interval. (TIF) [file ppat.1004869.s008.tif]
