# Supplementary material for: Host Transcriptional Response to Influenza and Other Acute Respiratory Viral Infections – A Prospective Cohort Study
Source: PLoS Pathog. 2015 Jun 12;11(6):e1004869. doi: 10.1371/journal.ppat.1004869 (PMC4466531; doi:10.1371/journal.ppat.1004869)
Supplement: S1 Table — (DOCX) [file ppat.1004869.s009.docx]

**Table S1 A List of the Transcript Probes that Were Plotted in the Time-course Expression Heatmaps Contrasting ARIs and Baseline**

Acute phase genes:

| Row No. | NuID | Gene Symbol | Gene Name |
| --- | --- | --- | --- |
| 1 | oUL016nU_reU_ae0_Y | IFI27 | interferon, alpha-inducible protein 27 |
| 2 | ijfUpc6MjSik0eKUok | IFITM3 | interferon induced transmembrane protein 3 |
| 3 | N4YTYufXzonoklZcgU | IRF7 | interferon regulatory factor 7 |
| 4 | TpnrGwMhQ5Lyunf1CE | OAS3 | 2'-5'-oligoadenylate synthetase 3, 100kDa |
| 5 | ukoevq3qjc6TUIS4R0 | OASL | 2'-5'-oligoadenylate synthetase-like |
| 6 | 0IiH14JJlUUJ0Il01k | OAS1 | 2'-5'-oligoadenylate synthetase 1, 40/46kDa |
| 7 | 3Aq_iknpEIp4peng8U | OAS1 | 2'-5'-oligoadenylate synthetase 1, 40/46kDa |
| 8 | oid6lfUuflFcTHf7ek | OAS2 | 2'-5'-oligoadenylate synthetase 2, 69/71kDa |
| 9 | o3qVxJeiBeiDXEVEgU | XAF1 | XIAP associated factor 1 |
| 10 | 0B2unl2eOJ63mNeDqo | IFI6 | interferon, alpha-inducible protein 6 |
| 11 | H5lhjlSDUgf7c0d1UE | IFI6 | interferon, alpha-inducible protein 6 |
| 12 | iqLR_Of0rw0ic6ucSk | EPSTI1 | epithelial stromal interaction 1 (breast) |
| 13 | WneIYOc6nkjh98ij_U | IFI44 | interferon-induced protein 44 |
| 14 | HFS7.KJOLlu7ubXtok | MX1 | myxovirus (influenza virus) resistance 1, interferon-inducible protein p78 (mouse) |
| 15 | 0nhUk0lEd6voA7_Xvo | IFIT2 | interferon-induced protein with tetratricopeptide repeats 2 |
| 16 | EEDSXrRSf9oEkiESKk | IFIT3 | interferon-induced protein with tetratricopeptide repeats 3 |
| 17 | fAEA0FqFUn.SB5KgEk | IFIT3 | interferon-induced protein with tetratricopeptide repeats 3 |
| 18 | NZ6BfqQoIO4kIsiIuU | RSAD2 | radical S-adenosyl methionine domain containing 2 |
| 19 | Tpa8gVuHs93V_jFXOU | HERC5 | hect domain and RLD 5 |
| 20 | 9lUF6j54kqCf5O_dwo | IFIT3 | interferon-induced protein with tetratricopeptide repeats 3 |
| 21 | fSohbtLKju5enuxurs | LY6E | lymphocyte antigen 6 complex, locus E |
| 22 | rupwjK1x5AUVdTP1sU | IFI44L | interferon-induced protein 44-like |
| 23 | uF4JJC4mp6Ku5KGF70 | ISG15 | ISG15 ubiquitin-like modifier |
| 24 | N4OCV6LHOJqV4h6eeE | IFIT1 | interferon-induced protein with tetratricopeptide repeats 1 |
| 25 | 055155VOJ7lC7lSp5M | MT1A | metallothionein 1A |
| 26 | 0Vb3Klul.liHskiAGw | HES4 | hairy and enhancer of split 4 (Drosophila) |
| 27 | lkoEEjkoEp5EnSRBPU | GBP5 | guanylate binding protein 5 |
| 28 | ivdSKCuggFOpKLKD4s | GBP1 | guanylate binding protein 1, interferon-inducible |
| 29 | Nk_t6ULcQ7VDNChBRU | GBP1 | guanylate binding protein 1, interferon-inducible |
| 30 | ikRLn9bpHkxDeKXXd0 | STAT1 | signal transducer and activator of transcription 1, 91kDa |
| 31 | BhFLTHp0qVFnDl8OA0 | TRIM22 | tripartite motif containing 22 |
| 32 | 6X_RJNfoqj2Qukle_M | PARP9 | poly (ADP-ribose) polymerase family, member 9 |
| 33 | 3XUk9LUqolGgFO9_fA | UBE2L6 | ubiquitin-conjugating enzyme E2L 6 |
| 34 | TpXkSunv37e87SKBLU | STAT2 | signal transducer and activator of transcription 2, 113kDa |
| 35 | uUK90R6Xqfq5UzKK3s | IFI35 | interferon-induced protein 35 |
| 36 | lUECOC9XNxoCk4eopU | LAP3 | leucine aminopeptidase 3 |
| 37 | 36hSSRC9Xt9OqYszhU | SERPING1 | serpin peptidase inhibitor, clade G (C1 inhibitor), member 1 |
| 38 | 3t9fhRTT15ePIqfXTo | SAMD9L | sterile alpha motif domain containing 9-like |
| 39 | cl2ge4RVTfYfSLffUI | IRF7 | interferon regulatory factor 7 |
| 40 | ipfoT8uHvJa1f0jUuk | MX2 | myxovirus (influenza virus) resistance 2 (mouse) |
| 41 | TSKXWS0XDR3XXfXvU8 | IL1RN | interleukin 1 receptor antagonist |
| 42 | upH6Ifs4COnuXeXt1U | IL1RN | interleukin 1 receptor antagonist |
| 43 | 6suzRLLJdSv18KhBNc | TNFSF10 | tumor necrosis factor (ligand) superfamily, member 10 |
| 44 | fcZTqE3D0iggrU7f6o | TNFSF13B | tumor necrosis factor (ligand) superfamily, member 13b |
| 45 | rpB1LSBJIDCblvSrUg | TNFSF13B | tumor necrosis factor (ligand) superfamily, member 13b |
| 46 | TY47U6f7qCMe6I4n1I | TNFAIP6 | tumor necrosis factor, alpha-induced protein 6 |
| 47 | fp75V5VfrXlXIxJL8w | IFITM1 | interferon induced transmembrane protein 1 (9-27) |
| 48 | xqk3l6LxfU06cXnuv4 | ZBP1 | Z-DNA binding protein 1 |
| 49 | fb3eXRMnxCUnogM6x0 | EIF2AK2 | eukaryotic translation initiation factor 2-alpha kinase 2 |
| 50 | iJedp7O.dkZ0Vn0uac | PRIC285 | peroxisomal proliferator-activated receptor A interacting complex 285 |
| 51 | 0PtPXs9FbUhfvRHdE4 | CX3CR1 | chemokine (C-X3-C motif) receptor 1 |
| 52 | fet10n1c1MykSIpJP4 | SIGLEC14 | sialic acid binding Ig-like lectin 14 |
| 53 | 09kidveWWSQwJ1.lmA | TYMP | thymidine phosphorylase |
| 54 | KZTRJ4H74jVbvHlzSQ | STAT1 | signal transducer and activator of transcription 1, 91kDa |
| 55 | ix1KUCgkUiUOgfjpXA | STAT1 | signal transducer and activator of transcription 1, 91kDa |
| 56 | fr.cFeEaHuTH9XTU54 | CEACAM1 | carcinoembryonic antigen-related cell adhesion molecule 1 (biliary glycoprotein) |
| 57 | xKXCKf3Uoo8n6L3cx0 | CEACAM1 | carcinoembryonic antigen-related cell adhesion molecule 1 (biliary glycoprotein) |
| 58 | 6KSl0.TRkk7kpegiQI | WARS | tryptophanyl-tRNA synthetase |
| 59 | BLXpdZI59P_Ffp5LoI | WARS | tryptophanyl-tRNA synthetase |
| 60 | HU6l.0SqESn3wBBWn0 | GBP4 | guanylate binding protein 4 |
| 61 | WguiinKqUS_efTwTIo | PARP14 | poly (ADP-ribose) polymerase family, member 14 |
| 62 | W2teeilGrlER2tWE44 | UBE2L6 | ubiquitin-conjugating enzyme E2L 6 |
| 63 | Qkl1OrSbvUIoBbBfkc | FCGR1B | Fc fragment of IgG, high affinity Ib, receptor (CD64) |
| 64 | lF4JSEqSKbWeEDR1Tg | TMEM140 | transmembrane protein 140 |
| 65 | WqenuuGedD9eu59oXw | FFAR2 | free fatty acid receptor 2 |
| 66 | HlbQueVeXu3q.e_nv8 | OTOF | otoferlin |
| 67 | odbdZwAxgDyUpuumJE | XAF1 | XIAP associated factor 1 |
| 68 | oqeOni_zvHB_leHr7k | TMEM123 | transmembrane protein 123 |
| 69 | 9Oe3u6BCbZDih3cShU | PLAC8 | placenta-specific 8 |
| 70 | cwpXkeAOQnSmWutvuE | PLAC8 | placenta-specific 8 |
| 71 | 6h9R5TXVCqVA9.S6cU | CXCL10 | chemokine (C-X-C motif) ligand 10 |
| 72 | cSj1OhRehCQFQHWCH4 | CCL2 | chemokine (C-C motif) ligand 2 |
| 73 | Ti7MlU37rB_efeR9M0 | LAMP3 | lysosomal-associated membrane protein 3 |
| 74 | HUrVUiUN5Vd3Od_B9w | OASL | 2'-5'-oligoadenylate synthetase-like |
| 75 | WeyXq7G.R0L3XHih34 | NCOA7 | nuclear receptor coactivator 7 |
| 76 | rXGgNcHFHpKAeTN968 | USP18 | ubiquitin specific peptidase 18 |
| 77 | EpBIouKLnnsjhBdT3M | SAT1 | spermidine/spermine N1-acetyltransferase 1 |
| 78 | QdQghd3XurkPge9Nb0 | NT5C3 | 5'-nucleotidase, cytosolic III |
| 79 | Np5CejE6D0kR.3V3x4 | SAMD9 | sterile alpha motif domain containing 9 |
| 80 | T03kDulKfqgQ465ECk | IFIH1 | interferon induced with helicase C domain 1 |
| 81 | ZSVvWSocin9p._oSQc | ISG20 | interferon stimulated exonuclease gene 20kDa |
| 82 | fSUs9G5SQSFx1H6cV4 | FAM46A | family with sequence similarity 46, member A |
| 83 | o3aWaFalm7iZn_Rdc0 | NA | NA |
| 84 | N6FHU_U3F50FeGl30Y | SCO2 | SCO cytochrome oxidase deficient homolog 2 (yeast) |
| 85 | Wk6rmu6qk7nl77zq.8 | BST2 | bone marrow stromal cell antigen 2 |
| 86 | op3vv4MBuBuBUpoKhU | PARP10 | poly (ADP-ribose) polymerase family, member 10 |
| 87 | utE_0gSunnu65TYtXk | PARP12 | poly (ADP-ribose) polymerase family, member 12 |
| 88 | EUAD7d6Qy8X1SMStVU | OAS2 | 2'-5'-oligoadenylate synthetase 2, 69/71kDa |
| 89 | 6V5WK54VD2eUAi7Q0g | SPATS2L | spermatogenesis associated, serine-rich 2-like |
| 90 | HdefXV5T01eVd1OCfg | OAS1 | 2'-5'-oligoadenylate synthetase 1, 40/46kDa |
| 91 | iqt.idQAMBH1.ioiRE | NA | NA |
| 92 | TR7dIiv9e52V793Sgk | CECR1 | cat eye syndrome chromosome region, candidate 1 |
| 93 | 6lPl9z1xF.1yqnrVp0 | NAGK | N-acetylglucosamine kinase |
| 94 | x6CNIVJ18V_3lL7FKk | IFI30 | interferon, gamma-inducible protein 30 |
| 95 | BUnS0JZROVEF0Uiog8 | FCN1 | ficolin (collagen/fibrinogen domain containing) 1 |
| 96 | Q1T_A_emeelo5P.i.s | MAFB | v-maf musculoaponeurotic fibrosarcoma oncogene homolog B (avian) |
| 97 | xhqv9fnXlKPALU4v34 | CD68 | CD68 molecule |
| 98 | ulT0A438W6XlU5861U | DHRS9 | dehydrogenase/reductase (SDR family) member 9 |
| 99 | T1aLrJnt164KLP7RHo | TIMM10 | translocase of inner mitochondrial membrane 10 homolog (yeast) |
| 100 | fctt136XSSd9XvfHr4 | PHF11 | PHD finger protein 11 |
| 101 | 0R3_RHfu..nR7RPVJE | PHF11 | PHD finger protein 11 |
| 102 | lo4foRHV6R4It4Eel4 | HERC6 | hect domain and RLD 6 |
| 103 | 9qsESLlfOCi_iNeQoI | NA | NA |
| 104 | Kh15Vr0FcUn7rB8e0Q | FBXO6 | F-box protein 6 |
| 105 | ipV3uAE5UmkKUEJ6qM | NA | NA |
| 106 | QjpTeSFCCQldBIHg3E | DDX60L | DEAD (Asp-Glu-Ala-Asp) box polypeptide 60-like |
| 107 | WRQ4OLXyV7tL8V2OV8 | TRIM5 | tripartite motif containing 5 |
| 108 | 9K1yu4XjFFFf7pJFqc | TOR1B | torsin family 1, member B (torsin B) |
| 109 | cenynVkOoAQWipSJ8s | DHX58 | DEXH (Asp-Glu-X-His) box polypeptide 58 |
| 110 | igVgS9VeuSoulMrp1w | OAS2 | 2'-5'-oligoadenylate synthetase 2, 69/71kDa |
| 111 | Ko6INenlO6_OUlXUiA | CMPK2 | cytidine monophosphate (UMP-CMP) kinase 2, mitochondrial |
| 112 | BUX_PpYB0uPpEIsr5I | OAS2 | 2'-5'-oligoadenylate synthetase 2, 69/71kDa |
| 113 | onYjOL3kArrWTX6V3o | SCARB2 | scavenger receptor class B, member 2 |
| 114 | THcpENrtDZ3SnUue_0 | DDX60 | DEAD (Asp-Glu-Ala-Asp) box polypeptide 60 |
| 115 | QV59WEUl06DOQQdexU | SHISA5 | shisa homolog 5 (Xenopus laevis) |
| 116 | NX9EfunjuDnhYBOiNU | IFI16 | interferon, gamma-inducible protein 16 |
| 117 | fcV3S0U75If1e3op0U | SP110 | SP110 nuclear body protein |
| 118 | Eo.0e9wnVbhe64pgKg | SP110 | SP110 nuclear body protein |
| 119 | iXpUnp.XtUDSUuzDSU | PLSCR1 | phospholipid scramblase 1 |
| 120 | rfqvqsrIxQOAR.ShfU | PARP9 | poly (ADP-ribose) polymerase family, member 9 |
| 121 | osGi.IlKnjn_u6Ukd4 | TAP1 | transporter 1, ATP-binding cassette, sub-family B (MDR/TAP) |
| 122 | ir7h1CJIuKCFVCTIdo | ADAR | adenosine deaminase, RNA-specific |
| 123 | 0tbjDbteANdkRHp66I | IRF9 | interferon regulatory factor 9 |
| 124 | 0igIDypdd43dnN5q14 | CNDP2 | CNDP dipeptidase 2 (metallopeptidase M20 family) |
| 125 | orpKKuCPgCeXPm7zno | GIMAP8 | GTPase, IMAP family member 8 |
| 126 | 9uj4uUrk1CH9XV9SCk | NUB1 | negative regulator of ubiquitin-like proteins 1 |
| 127 | Trqpb5K7Hqe0qN.3MU | SLC27A3 | solute carrier family 27 (fatty acid transporter), member 3 |
| 128 | 0_DHz7_krTURH3lVHk | GIMAP4 | GTPase, IMAP family member 4 |
| 129 | BqUICQfiX6nDeniy0s | ELF1 | E74-like factor 1 (ets domain transcription factor) |
| 130 | 3TXj7nnrbf3V0iS4SQ | VAMP5 | vesicle-associated membrane protein 5 (myobrevin) |
| 131 | T4.6rJDUogrnoirg5Y | PSME2 | proteasome (prosome, macropain) activator subunit 2 (PA28 beta) |
| 132 | fIB26Kp7Wd666G8L2g | TYMP | thymidine phosphorylase |
| 133 | NoYII8YdcmX3lVSFMk | DRAP1 | DR1-associated protein 1 (negative cofactor 2 alpha) |
| 134 | 9iDnutZBFSNhB16q3g | LGALS9 | lectin, galactoside-binding, soluble, 9 |
| 135 | Z0g_kkQIAZXdeH7Puk | KPNB1 | karyopherin (importin) beta 1 |
| 136 | l4OKUNCFIIFHQkjVJU | MLKL | mixed lineage kinase domain-like |
| 137 | xl7UnV3VQgEE4OJB9I | GBP2 | guanylate binding protein 2, interferon-inducible |
| 138 | iX3qB7qexxL0p66.jk | FGL2 | fibrinogen-like 2 |
| 139 | BuOl._Or.vu37gQtvE | CHMP5 | chromatin modifying protein 5 |
| 140 | lklI6yJkjlRR4CLh.g | CASP1 | caspase 1, apoptosis-related cysteine peptidase (interleukin 1, beta, convertase) |
| 141 | 9WQr2P9P4klI6yJkjk | CASP1 | caspase 1, apoptosis-related cysteine peptidase (interleukin 1, beta, convertase) |
| 142 | QKXwn_hUKgB5OiGT9o | LMO2 | LIM domain only 2 (rhombotin-like 1) |
| 143 | NpOOkK_yXAnTR.7K64 | LYSMD2 | LysM, putative peptidoglycan-binding, domain containing 2 |
| 144 | cVRiAQn6Uc5zTQv1no | NA | NA |
| 145 | ueUBIkurgOtUq4TvI0 | NA | NA |
| 146 | ck23SVuVCCedOOnrPg | NA | NA |
| 147 | xtiLhfeODuueK786Qk | NA | NA |
| 148 | ZehBCdbgheiYeCCPak | RPS18 | ribosomal protein S18 |
| 149 | ugpIWI4NdlM55zvhRU | NA | NA |
| 150 | 9XqVQCJySDWQH.Q3dw | RPS6 | ribosomal protein S6 |
| 151 | TX1a0enlIB0_C6OODk | NA | NA |
| 152 | N7v5121NRa1UQviIu0 | RPS4X | ribosomal protein S4, X-linked |
| 153 | 9jlZFNZxVjVdNCuDjE | RPS4X | ribosomal protein S4, X-linked |
| 154 | 6kK7jT4d.Xqpd3Qo4o | NA | NA |
| 155 | BsHTVRlSqR6T7dJF7s | NA | NA |

Recovery phase genes:

| Row No. | NuID | Gene Symbol | Gene Name |
| --- | --- | --- | --- |
| 156 | fSCcSeUrTkYKiRbogg | IGLL1 | immunoglobulin lambda-like polypeptide 1 |
| 157 | ln3fXXnHenVIxRaID4 | NA | NA |
| 158 | Eeqdeed6dUrlI7hNS8 | NA | NA |
| 159 | ZZeqdeed69UrlI7lNY | NA | NA |
| 160 | ceU7eTeyiEi0U0ftqY | NA | NA |
| 161 | lQ.oBKrVNCvS6C6N6g | LOC652694 | similar to Ig kappa chain V-I region HK102 precursor |
| 162 | HhecR84SAQucRJ7rVE | IGJ | immunoglobulin J polypeptide, linker protein for immunoglobulin alpha and mu polypeptides |
| 163 | 6Uf5NQR30VF1TGQqjs | TXNDC5 | thioredoxin domain containing 5 (endoplasmic reticulum) |
| 164 | 3gMUcd42..R4Vrimqo | NA | NA |
| 165 | fmpZa4DFHHeNv.0eFY | RN28S1 | RNA, 28S ribosomal 1 |
| 166 | 0WleallrgMUcd42..Q | NA | NA |
| 167 | NR5EdUfXSYWpX3UXXk | NA | NA |
| 168 | KeepdX_ldn1RXGOzgM | SPRYD3 | SPRY domain containing 3 |
| 169 | 3dV1_3L9UT1AKqXqOc | DPYSL5 | dihydropyrimidinase-like 5 |
| 170 | EeL0tpX1644f1EaASg | NA | NA |
| 171 | rGapBVea5EpVe3osqM | NA | NA |
| 172 | HXXVd17FICCUQdNFog | NA | NA |
| 173 | KTd53R6oV11X1Red0w | TPRA1 | transmembrane protein, adipocyte asscociated 1 |
| 174 | ZRKj6InrhXLQoJ64V4 | LOC100131726 | HCC-related HCC-C11_v3 |
| 175 | ZPTXR1R0xWXV6EvV54 | RNF213 | ring finger protein 213 |
| 176 | HCiVRKCg96CM5e6NeE | SNCA | synuclein, alpha (non A4 component of amyloid precursor) |
| 177 | oLN7F5VR0k.a59X9Hg | SNCA | synuclein, alpha (non A4 component of amyloid precursor) |
| 178 | Td4X07XynudeUdxXhE | AHSP | alpha hemoglobin stabilizing protein |
| 179 | rpw5Xp0QsU_I16Hv14 | HBD | hemoglobin, delta |
| 180 | T.OKlGre72HSXSoYd4 | NA | NA |
| 181 | c57uUl4hr15e36q_ug | RBM38 | RNA binding motif protein 38 |
| 182 | BnWcV6qY_yd4Tej_B0 | SELENBP1 | selenium binding protein 1 |
| 183 | cW9Lul4ARTu5QvUvRk | EPB42 | erythrocyte membrane protein band 4.2 |
| 184 | 0kqjYT4hQjkbWfXwTo | TESC | tescalcin |
| 185 | Nfj31YjJRegEcrl5Uo | GYPC | glycophorin C (Gerbich blood group) |
| 186 | uqS5KEE0nR6SgLX74o | GYPC | glycophorin C (Gerbich blood group) |
| 187 | QpPFWhSSKdN.ktCSug | NA | NA |
| 188 | NpZXkX0mj_qPKd.ysE | HAGH | hydroxyacylglutathione hydrolase |
| 189 | HQF2pRCEUfVfxXoSSg | ASCC2 | activating signal cointegrator 1 complex subunit 2 |
| 190 | 9XkaCEIiyXdS.SQWco | CSDA | cold shock domain protein A |
| 191 | TNJCXUutQFJZe9lujE | NA | NA |
| 192 | TJ5Vfbv1e7_eyepO3U | SLC25A39 | solute carrier family 25, member 39 |
| 193 | Q_UqYppVDhI.V.IVJI | FBXO7 | F-box protein 7 |
| 194 | 0Ju3s.O7iieen5Lmbk | BCL2L1 | BCL2-like 1 |
| 195 | fhJC1FcH7xEkTFEr3o | DCAF12 | DDB1 and CUL4 associated factor 12 |
| 196 | 610HU3AeDfqiBUjeFA | NA | NA |
| 197 | HU0M0eXWu8X6HiUg7o | NA | NA |
| 198 | KntX6g6ldIoS59QsTc | STRADB | STE20-related kinase adaptor beta |
| 199 | Q0R16neBERlJ13eCYc | ALPL | alkaline phosphatase, liver/bone/kidney |
| 200 | NeHlSg0ILnuCnfmo6U | PI3 | peptidase inhibitor 3, skin-derived |
| 201 | QIPRV4rkp9ekgjrh5I | HBG1 | hemoglobin, gamma A |
| 202 | TIPRV4rkp9ekgjrh6I | HBG2 | hemoglobin, gamma G |
