# Supplementary material for: Host Transcriptional Response to Influenza and Other Acute Respiratory Viral Infections – A Prospective Cohort Study
Source: PLoS Pathog. 2015 Jun 12;11(6):e1004869. doi: 10.1371/journal.ppat.1004869 (PMC4466531; doi:10.1371/journal.ppat.1004869)
Supplement: S2 Table — (DOCX) [file ppat.1004869.s010.docx]

**Table S2 A List of the Transcript Probes that Were Plotted in the Time-course Expression Heatmaps Contrasting Influenza and Rhinovirus Illness**

| Row No. | NuID | Gene Symbol | Gene Name |
| --- | --- | --- | --- |
| 1 | oUL016nU_reU_ae0_Y | IFI27 | interferon, alpha-inducible protein 27 |
| 2 | NeHlSg0ILnuCnfmo6U | PI3 | peptidase inhibitor 3, skin-derived |
| 3 | oNQKXoEQ6x0AEyeXao | PYGL | phosphorylase, glycogen, liver |
| 4 | HdUvciQ3EnvzuK5UEU | CPD | carboxypeptidase D |
| 5 | 3UIToHu90KvO6pup3U | C20orf3 | chromosome 20 open reading frame 3 |
| 6 | WkuK2yX.K6CC4q5Ju0 | CAB39 | calcium binding protein 39 |
| 7 | WeHeDzQgTFxfiVnlN0 | AGTPBP1 | ATP/GTP binding protein 1 |
| 8 | EdUoLS4_3.6n1XUChc | RTN3 | reticulon 3 |
| 9 | HkEgFzXR5XVS3d8C_g | CDKN1B | cyclin-dependent kinase inhibitor 1B (p27, Kip1) |
| 10 | QNB1VClYiueTFGxSJQ | NARF | nuclear prelamin A recognition factor |
| 11 | 3lHUoQr0A68QSXcXtk | CD44 | CD44 molecule (Indian blood group) |
| 12 | lUpdd7h3qcXS.VT.pQ | SLC12A9 | solute carrier family 12 (potassium/chloride transporters), member 9 |
| 13 | HSV3JIm0uS27zVp.xI | TMCO3 | transmembrane and coiled-coil domains 3 |
| 14 | iJfSh.qU7jlCKdKB6Q | XRN2 | 5'-3' exoribonuclease 2 |
| 15 | B6kued8DXF9R0HVMQc | CD302 | CD302 molecule |
| 16 | fpSuSF_qgh.AUXL3VY | PTAFR | platelet-activating factor receptor |
| 17 | fnXu8QqIHT_I0t76T4 | ZNF217 | zinc finger protein 217 |
| 18 | 3ddSK3kR1H0Tlvh3RI | TBC1D14 | TBC1 domain family, member 14 |
| 19 | QXbOR3pefdS8fn7sIE | PDZD8 | PDZ domain containing 8 |
| 20 | Hp4rv_K5Ngu9QnuHxc | RBPJ | recombination signal binding protein for immunoglobulin kappa J region |
| 21 | 6RCMCsuu7dhVNJd0VE | STAT5B | signal transducer and activator of transcription 5B |
| 22 | T4Ue8_8f4f9IkRX13o | CD44 | CD44 molecule (Indian blood group) |
| 23 | oxei.DEuJ3JCopnl.4 | MAPK1 | mitogen-activated protein kinase 1 |
| 24 | B1lTjnlQacbcacQ93k | MAEA | macrophage erythroblast attacher |
| 25 | 9jjkvez8_57t61wuiU | ATP6V1A | ATPase, H+ transporting, lysosomal 70kDa, V1 subunit A |
| 26 | W1ASS5ebVnUaLfUgRc | RFX2 | regulatory factor X, 2 (influences HLA class II expression) |
| 27 | 03U10RfeX_151f8JSs | TNFRSF9 | tumor necrosis factor receptor superfamily, member 9 |
| 28 | Njm.HTcEw3hKl0kKiU | NA | NA |
| 29 | NVr0.zm5iC0usHnkqc | NPEPPS | aminopeptidase puromycin sensitive |
| 30 | cKgKgtuiKSoA68JJWk | CCNY | cyclin Y |
| 31 | HvhnQiSj1Un9b5EBLg | CDC123 | cell division cycle 123 homolog (S. cerevisiae) |
| 32 | ilet45VBHlfn_.1e0U | PPM1M | protein phosphatase, Mg2+/Mn2+ dependent, 1M |
| 33 | NTVXOp1SVfkV0_e9I8 | PPP2R4 | protein phosphatase 2A activator, regulatory subunit 4 |
| 34 | ftdG9UoqafRX2wUohA | TRIB2 | tribbles homolog 2 (Drosophila) |
| 35 | ZumFCGP95fIkKj0V1k | LAG3 | lymphocyte-activation gene 3 |
| 36 | Q0R16neBERlJ13eCYc | ALPL | alkaline phosphatase, liver/bone/kidney |
| 37 | 69eJXi6CX97l_V.lR0 | ZDHHC19 | zinc finger, DHHC-type containing 19 |
| 38 | lDOHPcCnuKU6rPrwvk | DSC2 | desmocollin 2 |
| 39 | oUpULbXQtpegupegic | NA | NA |
| 40 | cnXl1aSHeSlQttdC0k | NA | NA |
| 41 | KXWGm3UpVAaXWtrqfc | NA | NA |
| 42 | u7qWpDUhJKTCkUvFeQ | IL4R | interleukin 4 receptor |
| 43 | EureCJ_vkgH2q3ECko | NA | NA |
| 44 | TfWml3WpUgnXQtpd1I | NA | NA |
| 45 | HXRJod1GVJyd2XR5pc | NA | NA |
| 46 | itdC_l1UpUkGl3aaX8 | NA | NA |
| 47 | cl3khULbXgtpd1KVJ0 | NA | NA |
| 48 | fdeVJ1SaV6yFRB9eCU | NA | NA |
| 49 | HVnD0xdXXjv.VK8Q6U | GNAQ | guanine nucleotide binding protein (G protein), q polypeptide |
| 50 | xqVnZNV9CG.1xvxZx8 | ROPN1L | rhophilin associated tail protein 1-like |
| 51 | 3ntYFxQiRAkJOTVuHk | LTA4H | leukotriene A4 hydrolase |
| 52 | 91Z.tRpirdZ6xlpeSA | TSPO | translocator protein (18kDa) |
| 53 | QCvqXVph5ZKVC_14LY | NA | NA |
| 54 | oN6iUS4VJRenSnL1A8 | SIRPA | signal-regulatory protein alpha |
| 55 | BKdqrVZtUpUqo6q2Yg | PPAP2C | phosphatidic acid phosphatase type 2C |
| 56 | B_tpUSfXglCdVqVJ0o | NA | NA |
| 57 | rAgveQgoc5rkrprq5k | NTN3 | netrin 3 |
| 58 | rfraVEn14JQnValSdI | NA | NA |
| 59 | HlS.9W1ppd1qVIJ10I | NA | NA |
| 60 | HhaCQVfRIRiRNpAVzg | GAS7 | growth arrest-specific 7 |
| 61 | B1eVUDTSvXavwiC63A | ZFP106 | zinc finger protein 106 homolog (mouse) |
| 62 | 6d17u7XqUdxS4.SHVk | AMICA1 | adhesion molecule, interacts with CXADR antigen 1 |
| 63 | Wo9eLx7.vXVR59U9eI | FNIP1 | folliculin interacting protein 1 |
| 64 | l3OLNlqVKKW7ijVkMA | OPLAH | 5-oxoprolinase (ATP-hydrolysing) |
| 65 | To0eg1I2JkvTkQpVvg | DICER1 | dicer 1, ribonuclease type III |
| 66 | 6eoMJ4q54Hvqgq4eyU | LPGAT1 | lysophosphatidylglycerol acyltransferase 1 |
| 67 | oF4lVFaiS7Xuf3k58g | TSPO | translocator protein (18kDa) |
| 68 | 6kEmr.khnfdSWieoe8 | NA | NA |
| 69 | rUJULM6V56vF64cT9U | ZFP106 | zinc finger protein 106 homolog (mouse) |
| 70 | Q_.z_qeqiszjiSH3aA | CAV2 | caveolin 2 |
| 71 | xHuV66ICR4XtOVV0Bc | FURIN | furin (paired basic amino acid cleaving enzyme) |
| 72 | iWC39U9H3cgJ5QhIpI | PAK2 | p21 protein (Cdc42/Rac)-activated kinase 2 |
| 73 | 0Eh3fdSWieoe9KR6ik | NA | NA |
| 74 | HXVppXkrdSUigofTok | NA | NA |
| 75 | 3.d4LruoLFKoekglSk | DHTKD1 | dehydrogenase E1 and transketolase domain containing 1 |
| 76 | HffXKpduN4quuXH1Hs | PPP3CA | protein phosphatase 3, catalytic subunit, alpha isozyme |
| 77 | c56kxoCx4lTOH4VEKo | MRPL16 | mitochondrial ribosomal protein L16 |
| 78 | rde4qmtUuRI7.0L10s | TTC38 | tetratricopeptide repeat domain 38 |
| 79 | il0V5OcojoXt3MSSyM | GALM | galactose mutarotase (aldose 1-epimerase) |
| 80 | oOlWe61Xd7V1N7O73s | KIAA0319L | KIAA0319-like |
| 81 | cwpXkeAOQnSmWutvuE | PLAC8 | placenta-specific 8 |
| 82 | Trqpb5K7Hqe0qN.3MU | SLC27A3 | solute carrier family 27 (fatty acid transporter), member 3 |
| 83 | QejSIV5d7_FWR4eDAk | GPBAR1 | G protein-coupled bile acid receptor 1 |
| 84 | cZeO2oSV51QsQMi4Vs | MT1G | metallothionein 1G |
| 85 | ECu1ECuSIC6D3ak24k | NA | NA |
| 86 | xjlJ93dSrny_n_VFXQ | JUP | junction plakoglobin |
| 87 | HiCg3odVNVUUowC14U | C19orf66 | chromosome 19 open reading frame 66 |
| 88 | rowuonJSMCeSgMk9Sk | NA | NA |
| 89 | ltevVv3UXIewF3YqSo | CDKN1A | cyclin-dependent kinase inhibitor 1A (p21, Cip1) |
| 90 | oVLqP09XJuujRUOUgg | HESX1 | HESX homeobox 1 |
| 91 | 6lPl9z1xF.1yqnrVp0 | NAGK | N-acetylglucosamine kinase |
| 92 | WkEvu3I7SKTp6l1N3k | TRIM38 | tripartite motif containing 38 |
| 93 | cCKea4lD8iVAiVYqBc | CDKN1C | cyclin-dependent kinase inhibitor 1C (p57, Kip2) |
| 94 | fKOS0UHEB6XoLEtNGU | TTC21A | tetratricopeptide repeat domain 21A |
| 95 | i4uimBR4lCiesvG_1k | TCN1 | transcobalamin I (vitamin B12 binding protein, R binder family) |
| 96 | orpKKuCPgCeXPm7zno | GIMAP8 | GTPase, IMAP family member 8 |
| 97 | Hkie59ehuPV.6Ceheg | APOBEC3G | apolipoprotein B mRNA editing enzyme, catalytic polypeptide-like 3G |
| 98 | 6TTnqoj3SEdjo1FRPQ | APOBEC3G | apolipoprotein B mRNA editing enzyme, catalytic polypeptide-like 3G |
| 99 | BdXlXor7fQnuh96j_Q | DUSP5 | dual specificity phosphatase 5 |
| 100 | QovQuig56f4ldxHnr4 | KLHDC8B | kelch domain containing 8B |
| 101 | 6dUoDtfd6beflfeLeU | ZFYVE26 | zinc finger, FYVE domain containing 26 |
| 102 | TR7dIiv9e52V793Sgk | CECR1 | cat eye syndrome chromosome region, candidate 1 |
| 103 | WU.npH.DqThLv3g3i0 | ZNF684 | zinc finger protein 684 |
| 104 | 6aaey6nd9dfUW4VQXc | NA | NA |
| 105 | 0CBX4XqmwwI4XoVeVU | ASCL2 | achaete-scute complex homolog 2 (Drosophila) |
| 106 | 3l_kqRDhftgNRe0oZc | CST3 | cystatin C |
| 107 | 9eDwAN766WpkunTl7o | DUSP19 | dual specificity phosphatase 19 |
| 108 | fhrMp_N7fnz75VeZck | HAVCR2 | hepatitis A virus cellular receptor 2 |
| 109 | i5OVK7e3eniv_3P8S4 | PHACTR2 | phosphatase and actin regulator 2 |
| 110 | BnlSXq3rAoAsS.SpCA | SSB | Sjogren syndrome antigen B (autoantigen La) |
| 111 | lKE1EqyK_ru7Gou3ik | TMEM62 | transmembrane protein 62 |
| 112 | i7XuHlUSIwqlKKj4Ck | ABI3 | ABI family, member 3 |
| 113 | cVpQTQuH8yeQgO6zug | NA | NA |
| 114 | 343VLftfFT1C53lJV4 | TRIM26 | tripartite motif containing 26 |
| 115 | B0iRUYDid0C9ApOUgw | RBM43 | RNA binding motif protein 43 |
| 116 | 3rFaUE0Lh.MnkIDus4 | NA | NA |
| 117 | WxOHSTF565Ingj.oq0 | CD38 | CD38 molecule |
| 118 | ujVBgklTmuHgSkMSxo | PPM1K | protein phosphatase, Mg2+/Mn2+ dependent, 1K |
| 119 | WXSeYr_hfin6OKg.9U | NA | NA |
| 120 | BoO4.pLSrHCTq6gdeU | UBIAD1 | UbiA prenyltransferase domain containing 1 |
| 121 | 3V_qkyHl9foV9QLu6w | C1orf162 | chromosome 1 open reading frame 162 |
| 122 | xFt6e5xguAECrV0KVY | APOBEC3F | apolipoprotein B mRNA editing enzyme, catalytic polypeptide-like 3F |
| 123 | HhIZKavz.oqsfuk.14 | ADPRHL2 | ADP-ribosylhydrolase like 2 |
| 124 | cQKpNoguSee5eO6gSc | MT1E | metallothionein 1E |
| 125 | cgblN7AkdIKSU1cjvo | RASGRP3 | RAS guanyl releasing protein 3 (calcium and DAG-regulated) |
| 126 | QeeYeOUoQX91SOwEiI | MT1F | metallothionein 1F |
| 127 | EuVBB.H5cQa9EB6BKU | PNPT1 | polyribonucleotide nucleotidyltransferase 1 |
| 128 | BmIi0nkEoIVDoyMQv4 | RABGAP1L | RAB GTPase activating protein 1-like |
| 129 | N5ieS0l9G3pdLVbtLk | OSBPL5 | oxysterol binding protein-like 5 |
| 130 | xUU5cfv5_cXl54VF4U | UBQLNL | ubiquilin-like |
| 131 | KuovxRRyF5GoIPnAqI | NA | NA |
| 132 | cg3llXU33F3g6UV8hU | C2 | complement component 2 |
| 133 | KJLSlERT51QDofd5CU | APOBEC3F | apolipoprotein B mRNA editing enzyme, catalytic polypeptide-like 3F |
| 134 | WXuPXrpSzXo9dwjfl0 | NSF | N-ethylmaleimide-sensitive factor |
| 135 | reEHuCUV6nEgFEt9Uk | ST3GAL5 | ST3 beta-galactoside alpha-2,3-sialyltransferase 5 |
| 136 | 3gRRofXYRTQiQXoSJU | IDH2 | isocitrate dehydrogenase 2 (NADP+), mitochondrial |
| 137 | fn9CiKQYgL6SgLhlNU | SAT2 | spermidine/spermine N1-acetyltransferase family member 2 |
| 138 | NZT8Vw.CBJqh4V1uWI | CUL1 | cullin 1 |
| 139 | ZqSpvyx7dV.FJAXh9E | C16orf61 | chromosome 16 open reading frame 61 |
| 140 | KCBFeWFAiFPiSiCaLg | TMSB10 | thymosin beta 10 |
| 141 | 05J7B50ZQDp46op558 | C22orf28 | chromosome 22 open reading frame 28 |
| 142 | TwlKEILkDl3_CQ9Kco | NHLRC3 | NHL repeat containing 3 |
| 143 | xuPF55OALk6qFe5N7k | NRIP1 | nuclear receptor interacting protein 1 |
| 144 | Kf7KZTSUf8glNJS7vo | ARHGEF3 | Rho guanine nucleotide exchange factor (GEF) 3 |
| 145 | 9ogng5BAT4nJRLkl6U | PSMA5 | proteasome (prosome, macropain) subunit, alpha type, 5 |
| 146 | 6AoeVxP6XuUelBaA6w | ANKRD36B | ankyrin repeat domain 36B |
| 147 | 0xmuhxiF_GaW93oZac | MRPS34 | mitochondrial ribosomal protein S34 |
| 148 | ilNVTfVyESSE3iAn0k | NA | NA |
| 149 | W.knji5z0AKCrN6CWk | GPD2 | glycerol-3-phosphate dehydrogenase 2 (mitochondrial) |
| 150 | WVH3uJJyTf3fedelR4 | KIAA0226 | KIAA0226 |
| 151 | 9K5eeNRXBVi3kdBiik | NA | NA |
| 152 | HVIERN4hKFMPPslUUI | USPL1 | ubiquitin specific peptidase like 1 |
| 153 | 6vUivofs8rDAnoXqHY | BRD7 | bromodomain containing 7 |
| 154 | lvYRUsVca4gko4vQm0 | ERP29 | endoplasmic reticulum protein 29 |
| 155 | Heq434Id4goI44Ka64 | EXOSC9 | exosome component 9 |
| 156 | 3XURH31AKCRW_LX.io | NSF | N-ethylmaleimide-sensitive factor |
| 157 | 65HhOCl0K9SvkkpuKQ | SYTL3 | synaptotagmin-like 3 |
| 158 | ESGzW6XfijNCgeIlbk | NUP205 | nucleoporin 205kDa |
| 159 | cXfiegZeAeiXRgqeec | SLC3A2 | solute carrier family 3 (activators of dibasic and neutral amino acid transport), member 2 |
| 160 | 3Uqb_h1LHQhKdMmeAk | BRD7 | bromodomain containing 7 |
| 161 | N7R5zufUVmWotUetyA | TMEM219 | transmembrane protein 219 |
| 162 | Qmd6QFgCjh6GtXzpZM | IL4I1 | interleukin 4 induced 1 |
| 163 | 9XpQhOSRQoUkPQgo0g | NA | NA |
| 164 | llN8l9xEHutN30tORY | OR52K2 | olfactory receptor, family 52, subfamily K, member 2 |
| 165 | cuUI45Qqe4.XrqCAgo | NA | NA |
| 166 | Ql.de397vz06DVLzeo | SLFN11 | schlafen family member 11 |
| 167 | EriFFI_dKgdSglSDgY | NA | NA |
| 168 | KqnEoqxcPIS91ARIEQ | FAM70A | family with sequence similarity 70, member A |
| 169 | ioJIoIo4AO5S4n4tfo | NDC80 | NDC80 homolog, kinetochore complex component (S. cerevisiae) |
| 170 | feGKkd4jJHneqU3jRQ | CNOT4 | CCR4-NOT transcription complex, subunit 4 |
| 171 | inreqMnokfHSunrgOE | ERP29 | endoplasmic reticulum protein 29 |
| 172 | lFD0SoByIcae66wXkg | STAP1 | signal transducing adaptor family member 1 |
| 173 | K6Et_Oq_IF96olI4V0 | MRPL17 | mitochondrial ribosomal protein L17 |
| 174 | B6KklUShr84kRDzJ_8 | CAPN2 | calpain 2, (m/II) large subunit |
| 175 | o5FTwXNVF3lUU83Se0 | NA | NA |
| 176 | NtBIdeIoBVnzeBHlp4 | CASS4 | Cas scaffolding protein family member 4 |
| 177 | 6DE0YpSe7j94hcjiLU | GAPVD1 | GTPase activating protein and VPS9 domains 1 |
| 178 | NdfVN0.fHl8Z014rUU | SLC46A3 | solute carrier family 46, member 3 |
| 179 | EXNKU7KAe737z6yKpU | NA | NA |
| 180 | 3WLeo0ucAt9ioiPcVk | NA | NA |
| 181 | 3XUP9UDkNLsHKp7v3k | KPNA1 | karyopherin alpha 1 (importin alpha 5) |
| 182 | ieKk.S9dcUXQSh.tSE | TRIM39 | tripartite motif containing 39 |
| 183 | N1uDV8it7g_yvCBV5U | SEC62 | SEC62 homolog (S. cerevisiae) |
| 184 | EoGHR8MFKgUlAx7klk | MMGT1 | membrane magnesium transporter 1 |
| 185 | 9KcIjuHEl5V4e77VKk | CTSK | cathepsin K |
| 186 | ZrlfHNT1cxFe9.VITE | EPM2AIP1 | EPM2A (laforin) interacting protein 1 |
| 187 | 0k_6fdLp_Ek316_zuk | MMGT1 | membrane magnesium transporter 1 |
| 188 | u_M5UdFdhg3lZ.qe64 | UBE2G1 | ubiquitin-conjugating enzyme E2G 1 |
| 189 | 9YWnbPWNLZ9T7yTbEY | NA | NA |
| 190 | fR1MSzyuKOjmpntflo | EHD1 | EH-domain containing 1 |
| 191 | NTqUQN3ELl5zV3VHVQ | ERF | Ets2 repressor factor |
| 192 | 0EmU3dXiDAljJRddac | CAPZB | capping protein (actin filament) muscle Z-line, beta |
| 193 | ECX53vu175ad6X17h4 | RXRA | retinoid X receptor, alpha |
| 194 | oJ.ldUoV4r5.N3S_6U | USP34 | ubiquitin specific peptidase 34 |
| 195 | 6WKAX5VTR0k_R8jEg4 | PID1 | phosphotyrosine interaction domain containing 1 |
| 196 | il313gyFIZX9Hy9LlI | ATP2B4 | ATPase, Ca++ transporting, plasma membrane 4 |
| 197 | oZhXmpJnyh5E.E.VA4 | NA | NA |
| 198 | lStNQXoN8n_FVBfdWk | NA | NA |
| 199 | xyHx56EN5OpNFXVZOw | NA | NA |
| 200 | clCoASEUgfq_KkcUh4 | ATXN1 | ataxin 1 |
| 201 | uRl_S4fdt6i6keLXdI | CYB561D1 | cytochrome b-561 domain containing 1 |
| 202 | f84K6T64p7tS5LLqYk | TKT | transketolase |
| 203 | HtULP1JKCw7fXSXQUo | HSPBAP1 | HSPB (heat shock 27kDa) associated protein 1 |
| 204 | Q4qALJ1J0FN4oKFJCg | NA | NA |
| 205 | HRX00gOoWQuspLtX3k | CACNA2D3 | calcium channel, voltage-dependent, alpha 2/delta subunit 3 |
| 206 | ZR8Ey4XUvU1Oe2QuEo | NA | NA |
| 207 | xnlXiCIfDUJePscuk0 | PPP1CB | protein phosphatase 1, catalytic subunit, beta isozyme |
| 208 | rt._UgvAp7dQtXgdJI | ERP27 | endoplasmic reticulum protein 27 |
| 209 | 6tSCHdd4e8X_VQlFUA | SSH2 | slingshot homolog 2 (Drosophila) |
| 210 | xXLfX01_V3vpEpPN3k | PUM2 | pumilio homolog 2 (Drosophila) |
| 211 | iukJL3.SSP_VHPXeJ4 | CD93 | CD93 molecule |
| 212 | ZHq.pehJHj_6O6OqkY | VENTX | VENT homeobox |
| 213 | 9cOh3Hr_Ln3iejhYWU | VPS37C | vacuolar protein sorting 37 homolog C (S. cerevisiae) |
| 214 | xnTT3qL9TsunOS7oC4 | STXBP5 | syntaxin binding protein 5 (tomosyn) |
| 215 | ZbJ5tv2M0RXe7ll8fU | NA | NA |
| 216 | rNWwSR7oMRXwQvol6Y | SORL1 | sortilin-related receptor, L(DLR class) A repeats containing |
| 217 | EShicef6Jir9ef_L4U | CDK19 | cyclin-dependent kinase 19 |
| 218 | WSp9200fkm7ifUDCWU | LOC100129550 | hypothetical LOC100129550 |
| 219 | xl68XtOg55Lrr_UJ94 | RAB22A | RAB22A, member RAS oncogene family |
| 220 | NOLV7khS6kKSepSN0o | FAM168B | family with sequence similarity 168, member B |
| 221 | lpOLyoh4Is_yHsTuX0 | TP53INP1 | tumor protein p53 inducible nuclear protein 1 |
| 222 | oVVlLXURUB794_n.J8 | PRKDC | protein kinase, DNA-activated, catalytic polypeptide |
| 223 | opfFAkAirkIO6izt5Y | UBE4B | ubiquitination factor E4B |
| 224 | WpCXO7viJ90hzUXeT4 | NA | NA |
| 225 | QVJXp6lSZe73u8gK.w | RNF44 | ring finger protein 44 |
| 226 | cfvpYuiHrPdAXrOutE | NA | NA |
| 227 | fsnbXR63Eevlg.H7PU | MYH9 | myosin, heavy chain 9, non-muscle |
| 228 | KA.52CX0tb5oomJ6GU | NA | NA |
| 229 | Nee665U3z14x54l6q4 | SORL1 | sortilin-related receptor, L(DLR class) A repeats containing |
| 230 | Eg5j0Rf7ktaN0fppCo | CAT | catalase |
| 231 | fLF6oryO7v0p_i7OLo | TP53INP1 | tumor protein p53 inducible nuclear protein 1 |
| 232 | 6VaC_SJDe67e0yVBy0 | NA | NA |
| 233 | x21B4QlfkXl3Uo7cQA | NA | NA |
| 234 | x__CSUetd_.VeSGioU | ZNF746 | zinc finger protein 746 |
| 235 | rcoepWKuvxeRb4dLMs | MYH9 | myosin, heavy chain 9, non-muscle |
| 236 | xT3qHFuCXosqImL_ok | ARAP3 | ArfGAP with RhoGAP domain, ankyrin repeat and PH domain 3 |
| 237 | 9SSCdJIx6EFTVfLIpE | NA | NA |
| 238 | 3mxHd1KQUncUXTUg_k | PGLYRP1 | peptidoglycan recognition protein 1 |
| 239 | QElrAaDRs69.e6t7pE | DEF8 | differentially expressed in FDCP 8 homolog (mouse) |
| 240 | u3V.VN_H3oMlflAxSo | NA | NA |
| 241 | ErXh0AVLiqjv.UoQuA | EMR3 | egf-like module containing, mucin-like, hormone receptor-like 3 |
| 242 | ZpO5egcWLKJT9.sVeU | CCNY | cyclin Y |
| 243 | NJQUDVFAFC_lGIuF3o | SIGLEC10 | sialic acid binding Ig-like lectin 10 |
| 244 | 3enFUiovtGcS4unpf8 | IMPA2 | inositol(myo)-1(or 4)-monophosphatase 2 |
| 245 | f3y1RvSuIIXQKxXe1I | NA | NA |
| 246 | TSXd75dJ3UL1KfWtfI | CA4 | carbonic anhydrase IV |
| 247 | NZdB55V6_Dvd_MuehU | ULK1 | unc-51-like kinase 1 (C. elegans) |
| 248 | lhC1QjlICkt1QoVFOQ | PPM1F | protein phosphatase, Mg2+/Mn2+ dependent, 1F |
| 249 | imXQeeVevg73fjLnoU | ULK1 | unc-51-like kinase 1 (C. elegans) |
| 250 | HlSI1AiV.iRRkII9Nk | NA | NA |
| 251 | QSm126C4TbfwFebpDU | RPLP0 | ribosomal protein, large, P0 |
| 252 | Ex9UJ7_91EMp3QQO7o | EMR3 | egf-like module containing, mucin-like, hormone receptor-like 3 |
| 253 | 6vSOEmFl.09VWUXsdE | TBL1X | transducin (beta)-like 1X-linked |
