# Supplementary material for: Host Transcriptional Response to Influenza and Other Acute Respiratory Viral Infections – A Prospective Cohort Study
Source: PLoS Pathog. 2015 Jun 12;11(6):e1004869. doi: 10.1371/journal.ppat.1004869 (PMC4466531; doi:10.1371/journal.ppat.1004869)
Supplement: S3 Table — (DOCX) [file ppat.1004869.s011.docx]

**Table S3 Cell Lineage and Activation State Markers Selected from the Published Literatures for Computing the Cell Scores**

| **Lineage-specific expression** | **Gene symbol** | **Description** | **Type** |
| --- | --- | --- | --- |
| Lymphocyte | CD72 | CD72 molecule | Transmembrane receptor |
| Lymphocyte | CNFN | cornifelin | Other |
| Lymphocyte | CD22 | CD22 molecule | Transmembrane receptor |
| Lymphocyte | VPREB3 | pre-B lymphocyte gene 3 | Transmembrane receptor |
| Lymphocyte | CD8A | CD8a molecule | Transmembrane receptor |
| Lymphocyte | CD8B | CD8b molecule | Transmembrane receptor |
| Lymphocyte | LRRN3 | leucine rich repeat neuronal 3 | Other |
| Lymphocyte | HKDC1 | hexokinase domain containing 1 | Kinase |
| Lymphocyte | SPTBN1 | spectrin, beta, non-erythrocytic 1 | Other |
| Lymphocyte | TCEA3 | transcription elongation factor A (SII), 3 | Transcription regulator |
| Lymphocyte | ZFP36L2 | zinc finger protein 36, C3H type-like 2 | Transcription regulator |
| Lymphocyte | LDLRAP1 | low density lipoprotein receptor adaptor protein 1 | Transporter |
| Lymphocyte | TCL1A | T-cell leukemia/lymphoma 1A | Transcription regulator |
| Neutrophil | CMTM2 | CKLF-like MARVEL transmembrane domain containing 2 | Cytokine |
| Neutrophil | CSF3R | colony stimulating factor 3 receptor (granulocyte) | Transmembrane receptor |
| Neutrophil | FCGR3A | Fc fragment of IgG, low affinity IIIa receptor (CD16a) | Transmembrane receptor |
| Neutrophil | IL8RB | interleukin 8 receptor, beta | G-protein-coupled receptor |
| Neutrophil | KRT23 | keratin 23 (histone deacetylase inducible) | Other |
| Neutrophil | MANSC1 | MANSC domain containing 1 | Other |
| Neutrophil | P2RY13 | purinergic receptor P2Y, G-protein coupled, 13 | G-protein-coupled receptor |
| Neutrophil | PROK2 | prokineticin 2 | Other |
| Neutrophil | RGS18 | regulator of G-protein signaling 18 | Other |
| Neutrophil | S100P | S100 calcium binding protein P | Other |
| Neutrophil | TNFRSF10C | tumor necrosis factor receptor superfamily, member 10c | Transmembrane receptor |
| Neutrophil | VNN2 | vanin 2 | Enzyme |
| Neutrophil | GLT1D1 | glycosyltransferase 1 domain containing 1 | Enzyme |
| Neutrophil | MME | membrane metallo-endopeptidase | Peptidase |
| Monocyte | LTA4H | leukotriene A4 hydrolase | Enzyme |
| Monocyte | FCN1 | ficolin (collagen/fibrinogen domain containing)1 | Other |
| Monocyte | ARRB1 | Arrestin, beta 1 | Other |
| Monocyte | FUCA1 | fucosidase, alpha-L- 1, tissue | Enzyme |
| Monocyte | EPHB2 | EPH receptor B2 | Kinase |
| Monocyte | MERTK | c-mer proto-oncogene tyrosine kinase | Kinase |
| Monocyte | VSIG4 | V-set and immunoglobulin domain containing 4 | Other |
| NK cell | EPHA4 | EPH receptor A4 | Kinase |
| NK cell | ATP9A | ATPase, Class II, type 9A | Other |
| NK cell | GPR56 | G protein-coupled receptor 56 | G-protein-coupled receptor |
| NK cell | PRSS23 | protease, serine, 23 | Peptidase |
| NK cell | CYBRD1 | cytochrome b reductase 1 | Enzyme |
| Activated NK cell | FRMD3 | FERM domain containing 3 | Other |
| Activated NK cell | SP100 | SP100 nuclear antigen | Transcription regulator |
| Activated NK cell | FAM46A | family with sequence similarity 46, member A | Other |
| Activated NK cell | SAMD9 | sterile alpha motif domain containing 9 | Other |
| Activated NK cell | NEXN | nexilin (F actin binding protein) | Other |
| Activated NK cell | LIPA | lipase A, lysosomal acid, cholesterol esterase | Enzyme |
| Activated NK cell | RTP4 | receptor (chemosensory) transporter protein 4 | Other |
|  |  |  |  |
