# Supplementary material for: Host Transcriptional Response to Influenza and Other Acute Respiratory Viral Infections – A Prospective Cohort Study
Source: PLoS Pathog. 2015 Jun 12;11(6):e1004869. doi: 10.1371/journal.ppat.1004869 (PMC4466531; doi:10.1371/journal.ppat.1004869)
Supplement: S4 Table — (DOCX) [file ppat.1004869.s012.docx]

**Table S4 Transcripts Showed Evidence of Correlation between Gene Expression on Day 0 and the Antibody Response**

| Row No. | Gene Symbol | Gene Name |  | Correlation Coefficient |
| --- | --- | --- | --- | --- |
| 1 | LILRB4 | leukocyte immunoglobulin-like receptor, subfamily B (with TM and ITIM domains), member 4 | | 0.42 |
| 2 | FZD2 | frizzled family receptor 2 | | 0.41 |
| 3 | KCNMB1 | potassium large conductance calcium-activated channel, subfamily M, beta member 1 | | 0.41 |
| 4 | OAS1 | 2'-5'-oligoadenylate synthetase 1, 40/46kDa | | 0.37 |
| 5 | SIGLEC16 | sialic acid binding Ig-like lectin 16 (gene/pseudogene) | | 0.36 |
| 6 | GRN | granulin |  | 0.35 |
| 7 | NKIRAS2 | NFKB inhibitor interacting Ras-like 2 | | 0.34 |
| 8 | CD68 | CD68 molecule | | 0.34 |
| 9 | LILRA1 | leukocyte immunoglobulin-like receptor, subfamily A (with TM domain), member 1 | | 0.34 |
| 10 | ADPRH | ADP-ribosylarginine hydrolase | | 0.33 |
| 11 | FGD2 | FYVE, RhoGEF and PH domain containing 2 | | 0.33 |
| 12 | SAT2 | spermidine/spermine N1-acetyltransferase family member 2 | | 0.33 |
| 13 | BLVRA | biliverdin reductase A | | 0.33 |
| 14 | LILRB1 | leukocyte immunoglobulin-like receptor, subfamily B (with TM and ITIM domains), member 1 | | 0.33 |
| 15 | DECR1 | 2,4-dienoyl CoA reductase 1, mitochondrial | | 0.32 |
| 16 | NHLRC3 | NHL repeat containing 3 | | 0.32 |
| 17 | BARD1 | BRCA1 associated RING domain 1 | | 0.32 |
| 18 | ADAP2 | ArfGAP with dual PH domains 2 | | 0.32 |
| 19 | C2 | complement component 2 | | 0.32 |
| 20 | FSCN1 | fascin homolog 1, actin-bundling protein (Strongylocentrotus purpuratus) | | 0.32 |
| 21 | SIL1 | SIL1 homolog, endoplasmic reticulum chaperone (S. cerevisiae) | | 0.32 |
| 22 | IFI30 | interferon, gamma-inducible protein 30 | | 0.31 |
| 23 | LGALS3BP | lectin, galactoside-binding, soluble, 3 binding protein | | 0.31 |
| 24 | RGL1 | ral guanine nucleotide dissociation stimulator-like 1 | | 0.30 |
| 25 | GPBAR1 | G protein-coupled bile acid receptor 1 | | 0.30 |
| 26 | ATF5 | activating transcription factor 5 | | 0.30 |
| 27 | MTMR11 | myotubularin related protein 11 | | 0.29 |
| 28 | XAF1 | XIAP associated factor 1 | | 0.29 |
| 29 | SERTAD1 | SERTA domain containing 1 | | 0.29 |
| 30 | SDSL | serine dehydratase-like | | 0.29 |
| 31 | BRSK1 | BR serine/threonine kinase 1 | | 0.29 |
| 32 | C17orf87 | chromosome 17 open reading frame 87 | | 0.29 |
| 33 | P2RX4 | purinergic receptor P2X, ligand-gated ion channel, 4 | | 0.28 |
| 34 | C4orf33 | chromosome 4 open reading frame 33 | | 0.28 |
| 35 | C11orf67 | chromosome 11 open reading frame 67 | | 0.28 |
| 36 | PSMB2 | proteasome (prosome, macropain) subunit, beta type, 2 | | 0.28 |
| 37 | IFITM3 | interferon induced transmembrane protein 3 | | 0.28 |
| 38 | P2RY2 | purinergic receptor P2Y, G-protein coupled, 2 | | 0.28 |
| 39 | ZNF259 | zinc finger protein 259 | | 0.28 |
| 40 | NDUFA9 | NADH dehydrogenase (ubiquinone) 1 alpha subcomplex, 9, 39kDa | | 0.28 |
| 41 | ATP1B3 | ATPase, Na+/K+ transporting, beta 3 polypeptide | | 0.28 |
| 42 | SLC7A7 | solute carrier family 7 (amino acid transporter light chain, y+L system), member 7 | | 0.28 |
| 43 | GART | phosphoribosylglycinamide formyltransferase, phosphoribosylglycinamide synthetase, phosphoribosylaminoimidazole synthetase | | 0.27 |
| 44 | COQ10A | coenzyme Q10 homolog A (S. cerevisiae) | | 0.27 |
| 45 | CD14 | CD14 molecule | | 0.27 |
| 46 | CBR1 | carbonyl reductase 1 | | 0.27 |
| 47 | TPMT | thiopurine S-methyltransferase | | 0.27 |
| 48 | PARP3 | poly (ADP-ribose) polymerase family, member 3 | | 0.27 |
| 49 | TIPRL | TIP41, TOR signaling pathway regulator-like (S. cerevisiae) | | 0.27 |
| 50 | RIPK3 | receptor-interacting serine-threonine kinase 3 | | 0.27 |
| 51 | CD300C | CD300c molecule | | 0.27 |
| 52 | ANXA2 | annexin A2 |  | 0.27 |
| 53 | GNG5 | guanine nucleotide binding protein (G protein), gamma 5 | | 0.26 |
| 54 | FAM125B | family with sequence similarity 125, member B | | 0.26 |
| 55 | HIST1H4H | histone cluster 1, H4h | | 0.26 |
| 56 | SLC9A9 | solute carrier family 9 (sodium/hydrogen exchanger), member 9 | | 0.26 |
| 57 | SLC6A12 | solute carrier family 6 (neurotransmitter transporter, betaine/GABA), member 12 | | 0.26 |
| 58 | C19orf56 | chromosome 19 open reading frame 56 | | 0.26 |
| 59 | PLAC8 | placenta-specific 8 | | 0.26 |
| 60 | TRIP6 | thyroid hormone receptor interactor 6 | | 0.26 |
| 61 | DRAP1 | DR1-associated protein 1 (negative cofactor 2 alpha) | | 0.26 |
| 62 | KCTD14 | potassium channel tetramerisation domain containing 14 | | 0.26 |
| 63 | RUFY3 | RUN and FYVE domain containing 3 | | 0.25 |
| 64 | DENND1A | DENN/MADD domain containing 1A | | 0.25 |
| 65 | ASCL2 | achaete-scute complex homolog 2 (Drosophila) | | 0.25 |
| 66 | RBM45 | RNA binding motif protein 45 | | 0.25 |
| 67 | MYOF | myoferlin |  | 0.25 |
| 68 | PSMA5 | proteasome (prosome, macropain) subunit, alpha type, 5 | | 0.25 |
| 69 | SCO2 | SCO cytochrome oxidase deficient homolog 2 (yeast) | | 0.25 |
| 70 | C1orf162 | chromosome 1 open reading frame 162 | | 0.25 |
| 71 | SIGLEC14 | sialic acid binding Ig-like lectin 14 | | 0.25 |
| 72 | GNS | glucosamine (N-acetyl)-6-sulfatase | | 0.25 |
| 73 | CDKN1A | cyclin-dependent kinase inhibitor 1A (p21, Cip1) | | 0.25 |
| 74 | SLC43A3 | solute carrier family 43, member 3 | | 0.25 |
| 75 | MAD2L1BP | MAD2L1 binding protein | | 0.25 |
| 76 | ASGR2 | asialoglycoprotein receptor 2 | | 0.25 |
| 77 | GRIPAP1 | GRIP1 associated protein 1 | | 0.25 |
| 78 | TOMM40L | translocase of outer mitochondrial membrane 40 homolog (yeast)-like | | 0.25 |
| 79 | ATOX1 | ATX1 antioxidant protein 1 homolog (yeast) | | 0.25 |
| 80 | DEM1 | defects in morphology 1 homolog (S. cerevisiae) | | 0.25 |
| 81 | IL4I1 | interleukin 4 induced 1 | | 0.24 |
| 82 | MASTL | microtubule associated serine/threonine kinase-like | | 0.24 |
| 83 | ERP29 | endoplasmic reticulum protein 29 | | 0.24 |
| 84 | APOBEC3G | apolipoprotein B mRNA editing enzyme, catalytic polypeptide-like 3G | | 0.24 |
| 85 | SMS | spermine synthase | | 0.24 |
| 86 | STYXL1 | serine/threonine/tyrosine interacting-like 1 | | 0.24 |
| 87 | GAS6 | growth arrest-specific 6 | | 0.24 |
| 88 | HAVCR2 | hepatitis A virus cellular receptor 2 | | 0.24 |
| 89 | HIST2H2AA3 | histone cluster 2, H2aa3 | | 0.24 |
| 90 | SCAND1 | SCAN domain containing 1 | | 0.24 |
| 91 | YWHAE | tyrosine 3-monooxygenase/tryptophan 5-monooxygenase activation protein, epsilon polypeptide | | 0.24 |
| 92 | BAK1 | BCL2-antagonist/killer 1 | | 0.24 |
| 93 | SNRPB2 | small nuclear ribonucleoprotein polypeptide B | | 0.24 |
| 94 | NEXN | nexilin (F actin binding protein) | | 0.23 |
| 95 | HPSE | heparanase |  | 0.23 |
| 96 | IFI44L | interferon-induced protein 44-like | | 0.23 |
| 97 | ST3GAL5 | ST3 beta-galactoside alpha-2,3-sialyltransferase 5 | | 0.23 |
| 98 | CLDN23 | claudin 23 |  | 0.23 |
| 99 | RSPH9 | radial spoke head 9 homolog (Chlamydomonas) | | 0.23 |
| 100 | ZFYVE26 | zinc finger, FYVE domain containing 26 | | 0.23 |
| 101 | VAMP5 | vesicle-associated membrane protein 5 (myobrevin) | | 0.23 |
| 102 | SASH3 | SAM and SH3 domain containing 3 | | 0.23 |
| 103 | NFKBIE | nuclear factor of kappa light polypeptide gene enhancer in B-cells inhibitor, epsilon | | 0.23 |
| 104 | CCL2 | chemokine (C-C motif) ligand 2 | | 0.23 |
| 105 | ZNF358 | zinc finger protein 358 | | 0.23 |
| 106 | MT1F | metallothionein 1F | | 0.23 |
| 107 | TLR7 | toll-like receptor 7 | | 0.23 |
| 108 | SLC37A1 | solute carrier family 37 (glycerol-3-phosphate transporter), member 1 | | 0.23 |
| 109 | GSDMD | gasdermin D |  | 0.23 |
| 110 | C19orf66 | chromosome 19 open reading frame 66 | | 0.23 |
| 111 | FUT4 | fucosyltransferase 4 (alpha (1,3) fucosyltransferase, myeloid-specific) | | 0.23 |
| 112 | LILRB2 | leukocyte immunoglobulin-like receptor, subfamily B (with TM and ITIM domains), member 2 | | 0.22 |
| 113 | TNFAIP3 | tumor necrosis factor, alpha-induced protein 3 | | 0.22 |
| 114 | CACNA1E | calcium channel, voltage-dependent, R type, alpha 1E subunit | | 0.22 |
| 115 | SLC2A6 | solute carrier family 2 (facilitated glucose transporter), member 6 | | 0.22 |
| 116 | CSTF3 | cleavage stimulation factor, 3' pre-RNA, subunit 3, 77kDa | | 0.22 |
| 117 | SCARB2 | scavenger receptor class B, member 2 | | 0.22 |
| 118 | MT1A | metallothionein 1A | | 0.22 |
| 119 | HLA-A | major histocompatibility complex, class I, A | | 0.22 |
| 120 | EDEM2 | ER degradation enhancer, mannosidase alpha-like 2 | | 0.22 |
| 121 | SIGLEC1 | sialic acid binding Ig-like lectin 1, sialoadhesin | | 0.22 |
| 122 | LDLR | low density lipoprotein receptor | | 0.22 |
| 123 | AKR1A1 | aldo-keto reductase family 1, member A1 (aldehyde reductase) | | 0.22 |
| 124 | FUZ | fuzzy homolog (Drosophila) | | 0.22 |
| 125 | HIST2H2AA4 | histone cluster 2, H2aa4 | | 0.22 |
| 126 | CD40 | CD40 molecule, TNF receptor superfamily member 5 | | 0.22 |
| 127 | SUSD1 | sushi domain containing 1 | | 0.22 |
| 128 | HIST2H2AC | histone cluster 2, H2ac | | 0.21 |
| 129 | DRG1 | developmentally regulated GTP binding protein 1 | | 0.21 |
| 130 | SLC24A4 | solute carrier family 24 (sodium/potassium/calcium exchanger), member 4 | | 0.21 |
| 131 | LGALS9B | lectin, galactoside-binding, soluble, 9B | | 0.21 |
| 132 | IFI6 | interferon, alpha-inducible protein 6 | | 0.21 |
| 133 | ASPRV1 | aspartic peptidase, retroviral-like 1 | | 0.21 |
| 134 | FXYD6 | FXYD domain containing ion transport regulator 6 | | 0.21 |
| 135 | IFITM1 | interferon induced transmembrane protein 1 (9-27) | | 0.21 |
| 136 | CHRNB1 | cholinergic receptor, nicotinic, beta 1 (muscle) | | 0.21 |
| 137 | RBM43 | RNA binding motif protein 43 | | 0.21 |
| 138 | SEPT4 | septin 4 |  | 0.21 |
| 139 | MRPL17 | mitochondrial ribosomal protein L17 | | 0.21 |
| 140 | FAM21A | family with sequence similarity 21, member A | | 0.21 |
| 141 | CD86 | CD86 molecule | | 0.21 |
| 142 | SFT2D2 | SFT2 domain containing 2 | | 0.21 |
| 143 | TMEM60 | transmembrane protein 60 | | 0.21 |
| 144 | TICAM1 | toll-like receptor adaptor molecule 1 | | 0.21 |
| 145 | RNH1 | ribonuclease/angiogenin inhibitor 1 | | 0.21 |
| 146 | DDAH2 | dimethylarginine dimethylaminohydrolase 2 | | 0.21 |
| 147 | HES4 | hairy and enhancer of split 4 (Drosophila) | | 0.21 |
| 148 | HIST2H4B | histone cluster 2, H4b | | 0.21 |
| 149 | TCN2 | transcobalamin II | | 0.21 |
| 150 | TAP2 | transporter 2, ATP-binding cassette, sub-family B (MDR/TAP) | | 0.21 |
| 151 | RNF213 | ring finger protein 213 | | 0.21 |
| 152 | TYROBP | TYRO protein tyrosine kinase binding protein | | 0.20 |
| 153 | ZUFSP | zinc finger with UFM1-specific peptidase domain | | 0.20 |
| 154 | BCAS2 | breast carcinoma amplified sequence 2 | | 0.20 |
| 155 | CNOT4 | CCR4-NOT transcription complex, subunit 4 | | 0.20 |
| 156 | MYD88 | myeloid differentiation primary response gene (88) | | 0.20 |
| 157 | UNC93B1 | unc-93 homolog B1 (C. elegans) | | 0.20 |
| 158 | TFG | TRK-fused gene | | 0.20 |
| 159 | CD36 | CD36 molecule (thrombospondin receptor) | | 0.20 |
| 160 | ATL3 | atlastin GTPase 3 | | 0.20 |
| 161 | EPSTI1 | epithelial stromal interaction 1 (breast) | | 0.20 |
| 162 | SLC3A2 | solute carrier family 3 (activators of dibasic and neutral amino acid transport), member 2 | | 0.20 |
| 163 | TCERG1 | transcription elongation regulator 1 | | 0.20 |
| 164 | NPC2 | Niemann-Pick disease, type C2 | | 0.20 |
| 165 | POMP | proteasome maturation protein | | 0.20 |
| 166 | MARCH1 | membrane-associated ring finger (C3HC4) 1 | | 0.20 |
| 167 | EFCAB11 | EF-hand calcium binding domain 11 | | 0.20 |
| 168 | RIN2 | Ras and Rab interactor 2 | | 0.20 |
| 169 | FOXO3 | forkhead box O3 | | -0.48 |
| 170 | TP53INP1 | tumor protein p53 inducible nuclear protein 1 | | -0.41 |
| 171 | SIAH1 | seven in absentia homolog 1 (Drosophila) | | -0.39 |
| 172 | TXNDC12 | thioredoxin domain containing 12 (endoplasmic reticulum) | | -0.36 |
| 173 | CYBRD1 | cytochrome b reductase 1 | | -0.33 |
| 174 | ZNF398 | zinc finger protein 398 | | -0.32 |
| 175 | FAM107B | family with sequence similarity 107, member B | | -0.31 |
| 176 | ARAP3 | ArfGAP with RhoGAP domain, ankyrin repeat and PH domain 3 | | -0.30 |
| 177 | DBR1 | debranching enzyme homolog 1 (S. cerevisiae) | | -0.30 |
| 178 | APPL2 | adaptor protein, phosphotyrosine interaction, PH domain and leucine zipper containing 2 | | -0.30 |
| 179 | C12orf57 | chromosome 12 open reading frame 57 | | -0.29 |
| 180 | ASF1B | ASF1 anti-silencing function 1 homolog B (S. cerevisiae) | | -0.29 |
| 181 | GMCL1 | germ cell-less homolog 1 (Drosophila) | | -0.29 |
| 182 | CD302 | CD302 molecule | | -0.29 |
| 183 | CAMK2G | calcium/calmodulin-dependent protein kinase II gamma | | -0.28 |
| 184 | NELL2 | NEL-like 2 (chicken) | | -0.28 |
| 185 | SGK1 | serum/glucocorticoid regulated kinase 1 | | -0.28 |
| 186 | RALBP1 | ralA binding protein 1 | | -0.28 |
| 187 | IRS2 | insulin receptor substrate 2 | | -0.28 |
| 188 | ZNF746 | zinc finger protein 746 | | -0.28 |
| 189 | SRSF8 | serine/arginine-rich splicing factor 8 | | -0.27 |
| 190 | NOV | nephroblastoma overexpressed gene | | -0.27 |
| 191 | GPR183 | G protein-coupled receptor 183 | | -0.27 |
| 192 | SLC35E1 | solute carrier family 35, member E1 | | -0.26 |
| 193 | RAB5B | RAB5B, member RAS oncogene family | | -0.26 |
| 194 | CABIN1 | calcineurin binding protein 1 | | -0.26 |
| 195 | NECAB2 | N-terminal EF-hand calcium binding protein 2 | | -0.26 |
| 196 | NQO2 | NAD(P)H dehydrogenase, quinone 2 | | -0.25 |
| 197 | DPEP2 | dipeptidase 2 | | -0.25 |
| 198 | CMTM2 | CKLF-like MARVEL transmembrane domain containing 2 | | -0.25 |
| 199 | IDS | iduronate 2-sulfatase | | -0.25 |
| 200 | TMEM71 | transmembrane protein 71 | | -0.25 |
| 201 | RTN3 | reticulon 3 |  | -0.24 |
| 202 | DAPK2 | death-associated protein kinase 2 | | -0.24 |
| 203 | CERS2 | ceramide synthase 2 | | -0.24 |
| 204 | PMS2P4 | postmeiotic segregation increased 2 pseudogene 4 | | -0.23 |
| 205 | PIGX | phosphatidylinositol glycan anchor biosynthesis, class X | | -0.23 |
| 206 | EIF3L | eukaryotic translation initiation factor 3, subunit L | | -0.23 |
| 207 | LYRM7 | Lyrm7 homolog (mouse) | | -0.23 |
| 208 | MEF2D | myocyte enhancer factor 2D | | -0.23 |
| 209 | FNIP1 | folliculin interacting protein 1 | | -0.23 |
| 210 | CBX7 | chromobox homolog 7 | | -0.22 |
| 211 | S1PR4 | sphingosine-1-phosphate receptor 4 | | -0.22 |
| 212 | HAL | histidine ammonia-lyase | | -0.21 |
| 213 | EGLN2 | egl nine homolog 2 (C. elegans) | | -0.21 |
| 214 | NUP98 | nucleoporin 98kDa | | -0.21 |
| 215 | NR4A2 | nuclear receptor subfamily 4, group A, member 2 | | -0.21 |
| 216 | TMX4 | thioredoxin-related transmembrane protein 4 | | -0.21 |
| 217 | RNF216L | ring finger protein 216-like | | -0.21 |
| 218 | KPNA4 | karyopherin alpha 4 (importin alpha 3) | | -0.21 |
| 219 | FOXO1 | forkhead box O1 | | -0.21 |
| 220 | RPS11 | ribosomal protein S11 | | -0.21 |
| 221 | GLS | glutaminase |  | -0.21 |
| 222 | UBE2G1 | ubiquitin-conjugating enzyme E2G 1 | | -0.20 |
| 223 | SNRPN | small nuclear ribonucleoprotein polypeptide N | | -0.20 |
| 224 | RNF103 | ring finger protein 103 | | -0.20 |
| 225 | DHRS7 | dehydrogenase/reductase (SDR family) member 7 | | -0.20 |
| 226 | MAPK1 | mitogen-activated protein kinase 1 | | -0.20 |
| 227 | KLHL24 | kelch-like 24 (Drosophila) | | -0.20 |
| 228 | WLS | wntless homolog (Drosophila) | | -0.20 |
| 229 | SNURF | SNRPN upstream reading frame | | -0.20 |
